# Consensus Paper: Experimental Neurostimulation of the Cerebellum

**DOI:** 10.1007/s12311-019-01041-5

**Published:** 2019-06-04

**Authors:** Lauren N. Miterko, Kenneth B. Baker, Jaclyn Beckinghausen, Lynley V. Bradnam, Michelle Y. Cheng, Jessica Cooperrider, Mahlon R. DeLong, Simona V. Gornati, Mark Hallett, Detlef H. Heck, Freek E. Hoebeek, Abbas Z. Kouzani, Sheng-Han Kuo, Elan D. Louis, Andre Machado, Mario Manto, Alana B. McCambridge, Michael A. Nitsche, Nordeyn Oulad Ben Taib, Traian Popa, Masaki Tanaka, Dagmar Timmann, Gary K. Steinberg, Eric H. Wang, Thomas Wichmann, Tao Xie, Roy V. Sillitoe

**Affiliations:** 1 Department of Pathology and Immunology, Department of Neuroscience, Program in Developmental Biology, Baylor College of Medicine, Jan and Dan Duncan Neurological Research Institute of Texas Children’s Hospital, 1250 Moursund Street, Suite 1325, Houston, TX 77030 USA; 2 Neurological Institute, Department of Neurosurgery, Cleveland Clinic, 9500 Euclid Avenue, Cleveland, OH 44195 USA; 3 Department of Exercise Science, Faculty of Science, University of Auckland, Private Bag 92019, Auckland, 1142 New Zealand; 4 Department of Neurosurgery, Stanford University School of Medicine, 1201 Welch Road, MSLS P352, Stanford, CA 94305-5487 USA; 5 Department of Neurology, Emory University, Atlanta, GA 30322 USA; 6 Department of Neuroscience, Erasmus Medical Center, 3015 AA Rotterdam, Netherlands; 7 Human Motor Control Section, NINDS, NIH, Building 10, Room 7D37, 10 Center Dr MSC 1428, Bethesda, MD 20892-1428 USA; 8 Department of Anatomy and Neurobiology, University of Tennessee Health Science Center, 855 Monroe Ave, Memphis, TN 38163 USA; 9 NIDOD Department, Wilhelmina Children’s Hospital, University Medical Center Utrecht Brain Center, Utrecht, Netherlands; 10 School of Engineering, Deakin University, Geelong, VIC 3216 Australia; 11 Department of Neurology, College of Physicians and Surgeons, Columbia University, New York, NY 10032 USA; 12 Department of Neurology, Yale School of Medicine, Department of Chronic Disease Epidemiology, Yale School of Public Health, Center for Neuroepidemiology and Clinical Research, Yale School of Medicine, Yale University, New Haven, CT 06520 USA; 13 Service de Neurologie, CHU-Charleroi, 6000 Charleroi, Belgium; 14 Service des Neurosciences, Université de Mons, 7000 Mons, Belgium; 15 Graduate School of Health, Physiotherapy, University of Technology Sydney, PO Box 123, Broadway, Sydney, NSW 2007 Australia; 16 Department of Psychology and Neurosiences, Leibniz Research Centre for Working Environment and Human Factors, Dortmund, Germany; 17 Department of Neurology, University Medical Hospital Bergmannsheil, Bochum, Germany; 18 Service de Neurochirurgie, CHU Saint-Pierre, 1000 Bruxelles, Belgium; 19 Defitech Chair of Clinical Neuroengineering, Center for Neuroprosthetics (CNP) and Brain Mind Institute (BMI), Ecole Polytechnique Federale de Lausanne (EPFL), Sion, Switzerland; 20 Department of Physiology, Hokkaido University School of Medicine, Sapporo, 060-8638 Japan; 21 Department of Neurology, University Hospital Essen, University of Duisburg-Essen, Essen, Germany; 22 R281 Department of Neurosurgery, Stanfod University School of Medicine, 300 Pasteur Drive, Stanford, CA 94305 USA; 23 Yerkes National Primate Research Center, Emory University, Atlanta, GA 30322 USA; 24 Department of Neurology, University of Chicago, 5841 S. Maryland Avenue, MC 2030, Chicago, IL 60637-1470 USA

**Keywords:** Cerebellum, Neurostimulation, Neuromodulation, DBS, Non-invasive therapy, Optogenetics

## Abstract

The cerebellum is best known for its role in controlling motor behaviors. However, recent work supports the view that it also influences non-motor behaviors. The contribution of the cerebellum towards different brain functions is underscored by its involvement in a diverse and increasing number of neurological and neuropsychiatric conditions including ataxia, dystonia, essential tremor, Parkinson’s disease (PD), epilepsy, stroke, multiple sclerosis, autism spectrum disorders, dyslexia, attention deficit hyperactivity disorder (ADHD), and schizophrenia. Although there are no cures for these conditions, cerebellar stimulation is quickly gaining attention for symptomatic alleviation, as cerebellar circuitry has arisen as a promising target for invasive and non-invasive neuromodulation. This consensus paper brings together experts from the fields of neurophysiology, neurology, and neurosurgery to discuss recent efforts in using the cerebellum as a therapeutic intervention. We report on the most advanced techniques for manipulating cerebellar circuits in humans and animal models and define key hurdles and questions for moving forward.

## Introduction (L.N. Miterko, J. Beckinghausen, R.V. Sillitoe)

The cerebellum has emerged as a promising target for neurostimulation in various diseases. Invasive cerebellar stimulation in animal models reveals exciting possibilities for work in humans and provides major hope as a novel intervention for disease conditions that are severe and respond poorly to drug treatment. Likewise, non-invasive cerebellar stimulation has provided new treatment possibilities and serves to uncover the fundamental mechanisms for how the human brain can be modulated by exogenous stimulation. In this consensus paper, we discuss recent animal and human stimulation paradigms that targeted the cerebellum, and as a group we attempt to identify key successes and failures, which are critical for improvements in human therapy. We outline important hurdles and suggest possible ways to overcome them. Before discussing the experimental and therapeutic cerebellar stimulation techniques that have been employed in human conditions and animal models, we first revisit the basic anatomical structure, connectivity, and function of the mammalian cerebellum in order to fully appreciate the outcomes of its stimulation in health and disease.

The basic cellular composition of the cerebellum was determined well over a century ago [1] and was expanded upon in recent years by more modern techniques [2, 3]. The firing properties of the different classes of cerebellar neurons have been extricated by in vitro and in vivo recordings [4–6]. Its finer connectivity was unveiled at the level of microcircuits [7–14], patterns [15–18], and individual types of electrical and chemical synapses using genetics, molecular biology, anatomy, and electrophysiology [19–22]. It is therefore safe to say that the circuitry of the cerebellum has been and still is among the most heavily investigated structures in the entire nervous system.

Viewed from the surface, the outer structure of the cerebellum can be grossly divided into three main regions [2]. The middle portion is the vermis and is named for its worm-like appearance. On either side of the vermis is a region called the paravermis, which is not structurally distinct, but does contain dedicated circuits for executing specific behaviors. The most lateral portions of the cerebellum are adjacent to each paravermis and are known as the hemispheres. Examination of its surface also reveals what is perhaps the most recognizable feature of the cerebellum in mammals, its highly folded architecture. The adult cerebellum is anatomically segmented into distinct folds called lobules [23]. There are ten primary lobules that are separated from one another by a series of fissures [2]. Because each fissure extends to a specific depth in the cerebellum, each lobule develops with a unique shape. However, all lobules contain the same canonical microcircuit.

**Fig. 1:**
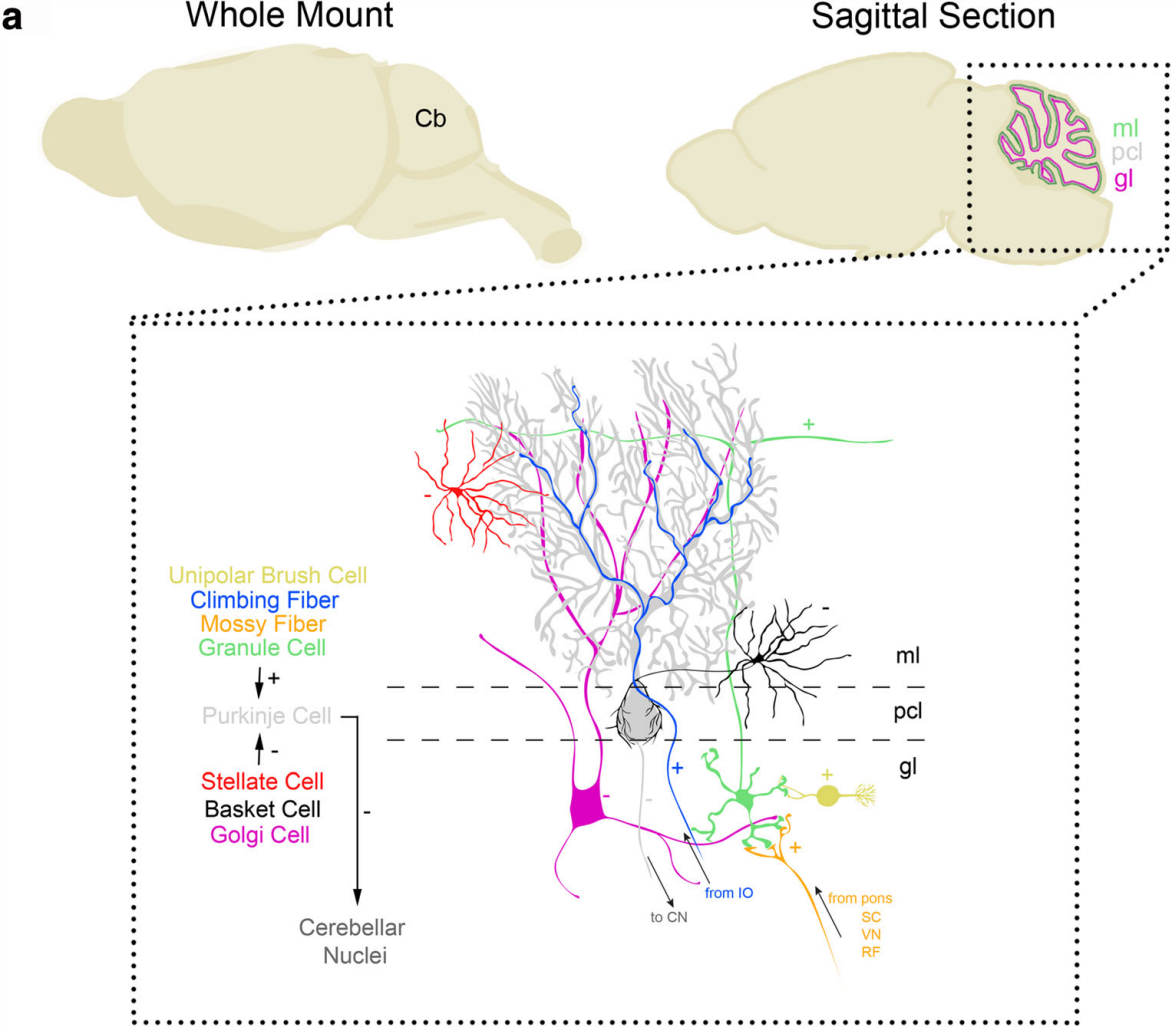
Schematic of the canonical cerebellar cortical circuit. **a** Cartoon drawing of the mouse brain (left) and a sagittal section illustrating the three layers of the cerebellar cortex (right). Schematic of the neurons in the cerebellar cortex (bottom, enlarged) illustrating the repeating basic circuitry that is comprised of Purkinje cells (gray), granule cells (green, with parallel fiber axons that bifurcate in the ml), climbing fiber afferents (blue), mossy fiber afferents (orange), stellate cell interneurons (red) and basket cell interneurons (black), Golgi cell interneurons (magenta), and unipolar brush cell interneurons (yellow). The excitatory synapses are labeled with a “+” and the inhibitory synapses with a “−” sign. The main output of the Purkinje cells is to the cerebellar nuclei, climbing fibers derive from inferior olive neurons, and mossy fibers come from a number of regions including the pontine nuclei, spinal cord, vestibular nuclei, and reticular formation. For simplicity, we have not shown the Lugaro cells or the candelabrum cells. Abbreviations: Cb = cerebellum, ml = molecular layer, pcl = Purkinje cell layer, gl = granular layer, cn = cerebellar nuclei, IO = inferior olive, SC = spinal cord, VN = vestibular nuclei, RF = reticular formation

The connectivity within the cerebellum is largely repeated through the structure, with each cell type forming stereotypical connections with its neighbors [1, 2, 24]. The cerebellum has three distinct layers, and each layer has distinct cell types (Fig. 1). The most superficial layer contains inhibitory stellate and basket cell interneurons and excitatory climbing and parallel fibers. These fibers and interneuron classes project onto Purkinje cells, which make up the middle layer called the Purkinje cell layer. The Purkinje cell layer also contains interneurons called candelabrum cells as well as specialized astrocytes called Bergmann glia. The Purkinje cells contribute to relaying the main computations of the cerebellar cortex onto downstream nuclei. The deepest layer is called the granular layer and it contains billions of small excitatory neurons called granule cells in addition to inhibitory Golgi cells, inhibitory Lugaro cells, mossy fibers that deliver excitatory signals to the granule cells, and a peculiar excitatory cell type called the unipolar brush cell. Unlike all other cell types that are found in all regions of the cerebellum, the unipolar brush cells are localized mainly in the vermis of lobules IX and X [9]. There are also modulatory “beaded” fibers that terminate in all layers of all lobules [25]. Below the three layers is the white matter that contains a dense network of fiber tracts. Embedded in this network are three bilateral pairs of cerebellar nuclei that are located on each side of the cerebellar midline. These nuclei contain specialized neurons that transmit the final output of the cerebellum, albeit that some types have also been shown to provide axon collaterals to the cerebellar cortex [11, 12, 26]. From medial to lateral, they are the fastigial, interposed, and dentate nuclei, all of which link the cerebellum to the rest of the brain and spinal cord [24]. The interposed nuclei can be divided into the anterior and posterior portions, which in primates are referred to as the emboliform and globose nuclei, respectively.

At the behavioral level, the output connections of the cerebellar nuclei are pertinent to our discussion of cerebellar stimulation in health and disease. The cerebellar nuclei project monosynaptic connections to the thalamus, vestibular nuclei, and inferior olive and in rodents have been shown to project to the red nucleus. The cerebellar nuclei were also recently shown to project directly to the locus coeruleus [27]. However, there are also polysynaptic short latency connections with critical structures, such as the basal ganglia [28, 29], in addition to other poorly defined, but likely functionally very important connections to the hypothalamus [30] and hippocampus [31]. There are also several other underappreciated cerebellar afferent pathways (e.g., cerebellar connections with the brainstem nuclei [32]) and efferent connections (e.g., from the periaqueductal gray [33]) that we will not discuss here, but suffice it to say that cerebellar stimulation almost certainly affects many more circuits than the output pathways to the thalamus. Furthermore, there have been marked advances in how we think about cerebellar-dependent behaviors. We are in unanimous and firm agreement that the cerebellum is required for motor behaviors ranging from coordination, posture, and balance, to learning and adaptation [34–37], although the exact mechanisms are far from clear. The role of the cerebellum is now beginning to be appreciated in behaviors previously thought to be strictly dedicated to brain regions that process non-motor functions, including emotion, language, and cognition [38–41]. This is an important issue to raise because all of the cerebellar neurostimulation paradigms must consider the large variety of behaviors that could potentially be affected.

Thanks to the advances in device engineering and technology, powerful pre-clinical animal models, and cutting-edge surgical methods, our view of the cerebellum in disease therapy has considerably evolved over the past two decades. Given that the future of brain stimulation holds promise for a treatment or an adjuvant treatment modality, we think this consensus paper is timely as the cerebellum is increasingly being implicated in a growing list of neurological and neuropsychiatric disorders.

However, although we think deeply towards the refinement of current approaches and techniques, as well as towards the potential for discovering future applications, we draw heavily upon the pioneering discoveries of functional neurosurgery and the initial findings of deep brain stimulation (DBS). Seventeen brief sections follow, with their topics spanning from different neurostimulation methods to proposed neurostimulation mechanisms. More specifically, we will discuss the current methods of neurotherapeutic brain stimulation such as DBS, experimental and clinically relevant methods such as transcranial magnetic stimulation (TMS), transcranial direct current stimulation (tDCS), and theta burst stimulation (TBS), and emerging methods of stimulation such as optogenetics, near-infrared, and magnetothermal DBS. These methods are considered in the context of cerebellar motor (coordination, balance, posture, learning) and non-motor (language, social cognition, emotion, literacy acquisition, attention) functions, as well as in the context of diseases such as ataxia, dystonia, essential tremor, Parkinson’s disease (PD), and stroke. The final sections of this consensus paper are dedicated to discussing the various advantages and disadvantages of cerebellar stimulation, and the potential mechanisms of action. Importantly, the systems level impact of electrical stimulation on the basic activity of the cerebellar cortex and cerebellar nuclei as well as the zonal organization of the cerebellum are considered. We also discuss the potential molecular impact of cerebellar stimulation.

## Origins of Cerebellar Stimulation

### Rationale for Cerebellar Stimulation (T. Wichmann, M.R. DeLong)

Current DBS approaches to treating movement disorders remain largely concentrated on efforts to influence basal ganglia and thalamic networks. The basal ganglia has been the favored DBS target to date because of the fact that link(s) between activity changes in specific basal ganglia circuits and movement disorders are relatively well-established. For example, one of the key insights in “systems” basal ganglia research within the last decades was the finding that the basal ganglia, thalamus, and cerebral cortex are components of anatomical circuits with separate territories in the individual anatomical nodes of the networks for “motor,” ‘associative,” and “limbic” functions [42, 43]. The concept of functional specificity of segregated basal ganglia circuits provides a rationale for the neurosurgical targeting of specific networks for motor and non-motor disorders. The clinically most important application of this knowledge has been the use of ablative or stimulation treatment applied to the basal ganglia motor circuit as treatment for hypo- and hyperkinetic disorders [44].

However, targeting other nodes of the motor circuit, such as the cerebellum, may also provide benefits [45]. While the cerebellum may not be as strictly segregated as the basal ganglia into motor and non-motor territories, there are several reasons as to why the cerebellum and its efferents should be considered as potential DBS targets for movement disorders. One reason is, of course, that cerebellar abnormalities have been demonstrated to occur in movement disorders, in particular, essential or parkinsonian tremor [36, 46–48] as well as some forms of dystonia [49–52]. Secondly, there is already a history of attempts to treat movement disorders with cerebellar stimulation (see section by Wichmann and DeLong under “Cerebellar Stimulation in Humans: Clinical Applications”). Another reason to consider that modulating the activity of cerebellar targets may be useful in the treatment of movement disorders is the finding of recent anatomical studies in primates which showed that there are strong bidirectional subcortical connections between subnuclei of the basal ganglia and the cerebellum which could have significant pathophysiological relevance for movement disorders [36, 53, 54]. Thus, basal ganglia activity may influence the cerebellum via projections of the STN to pontine nuclei which then project to the cerebellum [53]. In turn, projections from the deep cerebellar nuclei may directly influence basal ganglia activity via afferents to thalamic nuclei that project to the basal ganglia (primarily the striatum [55]). These interactions likely involve γ-aminobutric acid (GABA), glycine, glutamate, and dopamine due to the cellular composition of the deep cerebellar nuclei (GABA, glycine, glutamate [56]) as well as the role dopamine has in facilitating movement, learning, and non-motor behaviors in the striatum and the cerebellum [57–59]. It is therefore conceivable that modulating one of these systems (basal ganglia or cerebellum) influences the other.

## Cerebellar Stimulation in Animal Models: Pre-clinical Studies

While stimulating the basal ganglia and thalamus has been fruitful for a number of diseases, patient responses typically vary and its implementation is restricted to treating the most severe cases. Therefore, finding an alternative target for stimulation that can normalize patient responses and extend its use in the clinic is a top priority for clinicans and scientists alike. To test whether the cerebellum should be considered as an alternate stimulation target, we turn to animal models of human motor disease to assess its efficacy.

## Deep Brain Stimulation in Animal Models

### Implementation of Cerebellar DBS in Animal Models (L.N. Miterko, J. Beckinghausen, A.Z. Kouzani, R.V. Sillitoe)

Throughout Europe, Asia, and the USA, DBS is widely used to treat both tremor and human dystonia. In the USA, DBS is under the approval of the Food and Drug Association (FDA) for its use in treating tremor and dystonia. The internal segment of the globus pallidus is traditionally the target for treating dystonia with DBS [60], but patients receiving this surgery are not always responsive to stimulation. Based on previous and recent experimental data, there is a compelling argument that the cerebellum should be considered as a bonafide locus that participates in dystonia [49, 52, 61]. Therefore, we hypothesize that perhaps the reason for unresponsive surgeries could be due to the stimulation site rather than the efficacy of DBS itself [62].

**Fig. 2:**
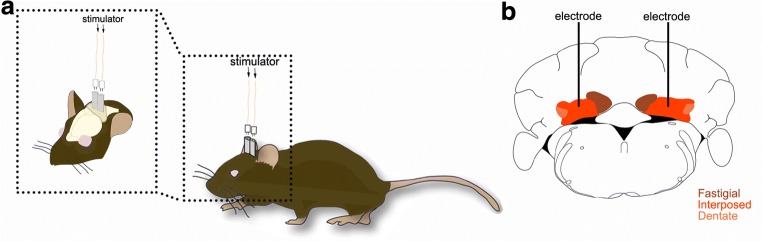
Deep brain stimulation of the mouse cerebellum. **a** Cartoon schematic of a mouse implanted with deep brain stimulation electrodes into the cerebellum. Even though this approach uses wires to connect the stimulator to the electrode port, there is enough flexibility for analysis in behaving animals. **b** Schematic of a tissue section cut through the mouse cerebellum illustrating the bilateral targeting of the bipolar stimulating electrodes to the interposed (middle) nucleus (red)

To test this hypothesis, we recently used the *Cre/LoxP* genetic approach to develop a new mouse model for testing the role of the cerebellum in dystonia [63]. By selectively silencing the glutamatergic output of olivocerebellar fibers, we were able to successfully induce a severe dystonia that initiated during development and continued throughout the life of the mice [63]. These data raised the possibility that perhaps our mice could serve as an ideal model for examining whether the cerebellar circuits for ongoing motion were optimal targets for DBS. For this reason, we targeted the interposed nuclei (Fig. 2), which project to several areas, such as the red nucleus and thalamus, through which they modulate movement. We used bilaterally implanted twisted bipolar electrodes, and in general the approach was inspired by the paradigms used for pre-clinical non-human primate studies and the treatment of human PD [64]. We reported immediate improvement in motor behavior with the alleviation of twisting postures and rigidity [63]. We also implanted DBS electrodes into the centrolateral nucleus of the thalamus, a region implicated in mediating the communication between the cerebellum and basal ganglia in dystonia [29]. In accordance with the idea of a “dystonia circuit,” high frequency stimulation of the centrolateral nucleus also improved movement in our mouse model of dystonia [63].

Our DBS results in rodent dystonia are promising for human therapy, but there are many questions that should be addressed if the cerebellum is to make it onto a shortlist of targets for this and other motor diseases. In the specific case of dystonia, in what circumstances should the cerebellum be considered for therapy? An alternative question, and perhaps not mutually exclusive, is when should globus pallidus stimulation not be the primary choice? Certainly, the neurologist and neurosurgeon have to assess each patient and their history, but even armed with an evaluation, these questions are still non-trivial to address. A major consideration in this regard is what type of dystonia the patient has, and if it is a genetic form, is there any indication that the mutant gene and its effects involve the cerebellum? This problem has yet to be solved, although for *Dyt1* at least there is some indication that the genetic pathway in the cerebellum is at fault [65].

There is a long history of cerebellar stimulation for dystonia-related behaviors [66–71], although due to much needed regulations, progress was unfortunately turbulent [72]. Still, much optimism has remained [73]. In accordance with this, our results showing that multiple motor features that are indicative of human dystonia are convincingly alleviated in our mouse model of dystonia support cerebellar stimulation for human therapy [63]. In addition, although we showed a specific utility of interposed stimulation in dystonia-like behavior, our study was not the first demonstration of using the cerebellar nuclei for motor repair in pre-clinical models. Elegant work from the Machado group (see section by Cooperrider and colleagues in this Consensus) consisted of inducing stroke in rats and then stimulating the dentate nucleus to improve motor outcome [74]. These studies are supported by optogenetic stimulation of the dentate, which also provides motor benefits ([75]; see section by Cheng and colleagues in this Consensus). More recently, Machado and colleagues have translated their findings from the rodent into humans, where they are currently testing in clinical trials if dentate nucleus stimulation improves motor function after stroke (see section by Cooperrider and colleagues in this Consensus). With the resurgence of cerebellar nuclei stimulation as a potential therapy, come many questions. The most pressing question is, what is the mechanism of action?

## Optogenetic Stimulation in Animal Models

### Cerebellar Optogenetics in Stroke Research (M.Y. Cheng, E.H. Wang, G.K. Steinberg)

Increasing brain activity can lead to the release of trophic factors, axonal sprouting, and myelination—all of which are beneficial for brain repair [76, 77]. Conventional brain stimulation techniques such as electrical stimulation, TMS and tDCS, allow direct manipulation of a region’s excitability and enhance recovery after stroke [78, 79]. However, these techniques may also induce undesirable side effects in addition to the potential functional gains. Various strategies have been used to improve functional outcomes after stroke such as stem cell therapies and pharmacological interventions [80, 81]. While cell therapy and drugs may catalyze endogenous repair processes, these approaches lack the necessary spatial resolution to precisely target specific areas.

To circumvent this, our laboratory employed optogenetics as a tool to selectively stimulate specific cell populations after stroke, enabling further targeting precision and the ability to disentangle heterogeneous stimulation effects. The brain has a remarkable capacity for plasticity after stroke, in both areas adjacent to the infarct (the peri-infarct) and the remotely connected regions [82]. Therefore, recovery from stroke likely requires re-mapping lost function onto surviving neural circuitry through structural and functional plasticity [82, 83]. Extensive studies have focused on changes in the peri-infarct region, including activation of an axonal sprouting program, cellular composition changes (astrocyte and microglia proliferation/migration), and neurophysiological properties [84, 85]. While some of these adaptations may exacerbate injury such as pro-inflammatory microglia activation [86], other changes such as increased neural excitability are positively correlated with good functional outcomes [87].

Increasing research efforts have focused on stroke-induced changes in remotely connected regions, including cortical areas in the contralesional hemisphere, thalamus, and the cerebellum, since stroke can disrupt neuronal function within minutes and extend this effect to connected areas [88, 89]. In particular, stroke can cause changes in the cortico-cerebellar system, resulting in depression of brain metabolism and function in the cerebellum; this is known as crossed cerebellar diaschisis [90]. In turn, this leads to dysfunction in both motor and non-motor functions, including balance, coordination, and visuospatial perception [90, 91]. Crossed cerebellar diaschisis has been reported as a potential prognosis indicator for stroke recovery [90].

Data from our laboratory and others have shown that increasing excitability of the ipsilesional primary motor cortex (iM1) after stroke is beneficial for recovery [81, 92]. Using optogenetic neuronal stimulation, we showed that repeated neuronal stimulation in iM1 promotes behavioral recovery in a stroke mouse model, with an associated increase in cerebral blood flow, neurovascular coupling response, and an increase in neurotrophins. Importantly, stimulated mice exhibited increased expression of the axonal growth associated protein 43 (GAP43), suggesting that stimulation-enhanced recovery may enhance structural plasticity [92]. Within the cerebellum, the lateral cerebellar nucleus (LCN) has emerged as a promising brain stimulation target. LCN is the largest of the four cerebellar nuclei in primates and sends major excitatory output to the motor, premotor, and somatosensory cortex via the dentato-thalamo-cortical pathway [93]. Post-stroke chronic electrical stimulations in the rat LCN have been shown to enhance stroke recovery, with an increased expression of markers for synaptogenesis and long-term potentiation [74]. Chronic LCN stimulations also increased neurogenesis selectively in glutamatergic neurons of the motor cortex [94].

We have recently demonstrated that selective neuronal stimulation in the contralesional LCN using optogenetic approaches resulted in robust and persistent recovery after stroke, as stroke mice maintained their improved performance even after cessation of stimulation for 2 weeks [75]. The persistent recovery suggests that repeated LCN stimulations may enhance structural plasticity. Examination of GAP43 expression further supports this speculation, as LCN stimulations significantly increased the plasticity marker, GAP43, in the ipsilesional somatosensory cortex, and its expression was positively correlated with improved functional outcomes [75]. The mechanisms of LCN stimulation-enhanced recovery likely involve multiple mechanisms, including activity-dependent molecules such as cfos and CREB, which are transcription factors that mediate an array of downstream genes involved in cell survival and synaptic plasticity [95]. High throughput next generation sequencing in LCN stimulation-induced axonal sprouted neurons can reveal important biological pathways underlying stimulation-induced recovery, which may provide potential drug targets for enhancing stroke recovery.

The cerebellar brain stimulation studies have highlighted LCN as a promising brain stimulation target. It is an anatomically small brain region that contains widespread projections to multiple brain regions, thus activating this single site has the potential to result in widespread brain activation [93]. Indeed, our indirect comparison suggests that stimulating the LCN can potentially be more efficacious than stimulating the motor cortex, as LCN-stimulated mice exhibited fast and robust recovery. Several clinical studies support the use of LCN stimulation in stroke patients. A recent study used probabilistic tractography to demonstrate that the dentate-thalamo-cortical tract was positively correlated to both general motor output and fine motor skills in chronic stroke patients, further highlighting the importance of the cerebellar dentate-thalamo-cortical circuit [96]. A recent case study reported that a woman with a cerebellar stroke exhibited improvements in cerebellar ataxia after DBS in the cerebellar LCN, further supporting the feasibility of LCN stimulation for stroke patients [97]. While using optogenetics to enhance stroke recovery is highly dependent on exogenous gene therapy being approved for use in clinical trials, our data further supports the stimulation of the cerebellar circuit to facilitate treatments for stroke recovery.

### Cerebellar Optogenetic Stimulation for Epilepsy (S.V. Gornati, F.E. Hoebeek)

Thus far, the diseases in which we discussed—e.g., dystonia and stroke—have been successfully treated in rodents by stimulating the cerebellar nuclei. Epilepsy is an additional neurological disease by which cerebellar stimulation holds promise. Epilepsy is a neurological disorder characterized by episodes of dysfunctional neuronal network activity. The seizures, which often come about due to hyper-synchronous neuronal firing [98], can be the result of many different causes: brain injury, stroke, genetic mutations, and birth defects [99]. Approximately ~ 30% of epilepsy patients do not respond adequately to anti-epileptic drugs and thus may need surgical resection of the seizure focus, or neurostimulation. Whereas vagal nerve stimulation is commonly used in refractory epilepsy patients [100], an increasing number of patients receive intracranial DBS [101]. However, the first brain region that was selected for DBS in epilepsy patients was the cerebellum, in effort to counter the hyperexcitability of thalamo-cortical pathways [102].

Landmark studies on the origin of (excessive) thalamo-cortical burst firing revealed that the balance between inhibition and excitation in thalamo-cortical networks is effective in setting the firing pattern of thalamo-cortical relay neurons by controlling the activation of low-threshold voltage-gated Ca^2+^ channels (as reviewed in ref [103]). Thus, by increasing the excitatory drive onto thalamic neurons, the burst-firing of thalamic relay neurons can be prevented. A recent study showed in epileptic mouse and rat models that membrane depolarization of thalamic relay neurons prevented burst-firing and thereby stopped generalized absence seizures [104]. Likewise, it has also been shown in various mouse models by pharmacological manipulation of the cerebellar nuclei neurons, which form numerous glutamatergic synapses throughout the thalamic complex, that increasing cerebellar nuclei firing frequency dampens the occurrence of generalized absence seizures. Notably, decreasing cerebellar nuclei firing potently increased such seizures, which contrasts initial hypotheses that increased inhibition of the cerebellar nuclei reduce seizures [105]. These findings underline the importance of gaining precise control over the cerebellar output for optimal therapeutic effects.

To ensure a temporally precise activation of inhibitory or excitatory inputs, optogenetic stimulation is a seemingly ideal tool. Optogenetics avoid the weakness of a-specific effects by electrical stimulation and the temporal resolution is sufficient to mimic endogenous activity patterns in most types of neurons [106]. Moreover, by expressing light-activated proteins like channelrhodopsin (ChR2) or halorhodopsin (HR) in specific cell types, optogenetics allow full control over action potential firing patterns. For instance, the expression of ChR2 in Purkinje cells, which can be induced using transgenic mutant mice, by in utero electroporation or by viral injections [107], allows precise control over action potential firing in their downstream target, the cerebellar nuclei [108] and thereby over cerebellar-evoked excitation or inhibition in the thalamus.

Optogenetic stimulation of the cerebellar cortex has so far been tested in two experimental studies. Krook-Magnuson and colleagues investigated the impact of on-demand optogenetic stimulation or inhibition of cerebellar Purkinje cells on seizures induced by intrahippocampal kainic acid injections [109]. The authors found that the seizure duration can be shortened upon activation of ChR2- or HR channels in both laterally and medially localized Purkinje cells, but that the seizure occurrence could only be dampened when the midline Purkinje cells were optogenetically excited. These findings indicate that the cerebellar cortical stimulation, which putatively stopped action potential firing in cerebellar nuclei neurons, revealed therapeutic effects on limbic seizures. In contrast, absence seizures occur more frequently upon pharmacological inhibition of cerebellar nuclei activity [105]. Instead, optogenetic excitation of cerebellar nuclei neurons consistently resulted in an abrupt stop of cerebral seizure activity. These findings on the impact of cerebellar manipulations on the various types of seizures indicate that cerebellar stimulation can have a widely varying effect on the seizure occurrence due to how diverse innervation onto thalamic nuclei is itself by cerebellar nuclei [110]. Indeed, also in the earlier reports on the effects of low- or high-frequency stimulation (10–200 Hz), it was noted that either seizure occurrence was dampened or enhanced (e.g., [111]).

One potential source for the variability in effects of cerebellar stimulation comes from the diverse anatomical connections that may be stimulated. Even though the cerebello-thalamic projection is mono-synaptic and purely glutamatergic [110], many cerebellar nuclei axons also project to inhibitory neurons in the zona incerta and anterior pretectal nucleus, which provide dense inhibitory input to thalamic nuclei [112, 113]. Thereby, the cerebellar impact on thalamic nuclei is most likely multi-phasic, in that an optogenetically induced increase in glutamate release from cerebellar nuclei axons in the thalamus may be followed by an increase in GABA. Although the impact of such feed-forward connections is currently unknown, we postulate that these speculative multi-phasic responses in thalamus evoked by cerebellar nuclei stimulation aid to stop thalamo-cortical oscillations by increasing the excitatory drive onto thalamic relay neurons and by desynchronizing thalamo-cortical activity. It remains to be investigated whether the impact of chronic, non-responsive stimulation paradigms are as effective as the responsive cerebellar cortical or cerebellar nuclei stimulation [105, 109]. Further research is also warranted to elucidate whether cerebellar photostimulation has a broad therapeutic effect against a variety of seizures and if optogenetics is feasibly translatable to humans. For long-term use in humans, implantable light probes must be operable and biocompatible as well as there must be efficient delivery, retainment, and expression of opsins in vivo to target cells with minimal adverse immune responses.

## Translating Cerebellar Stimulation to Humans

### Cerebellar DBS in Stroke: From Pre-clinical to Clinical Trials (J. Cooperrider, Kenneth B. Baker, A. Machado)

**Fig. 3:**
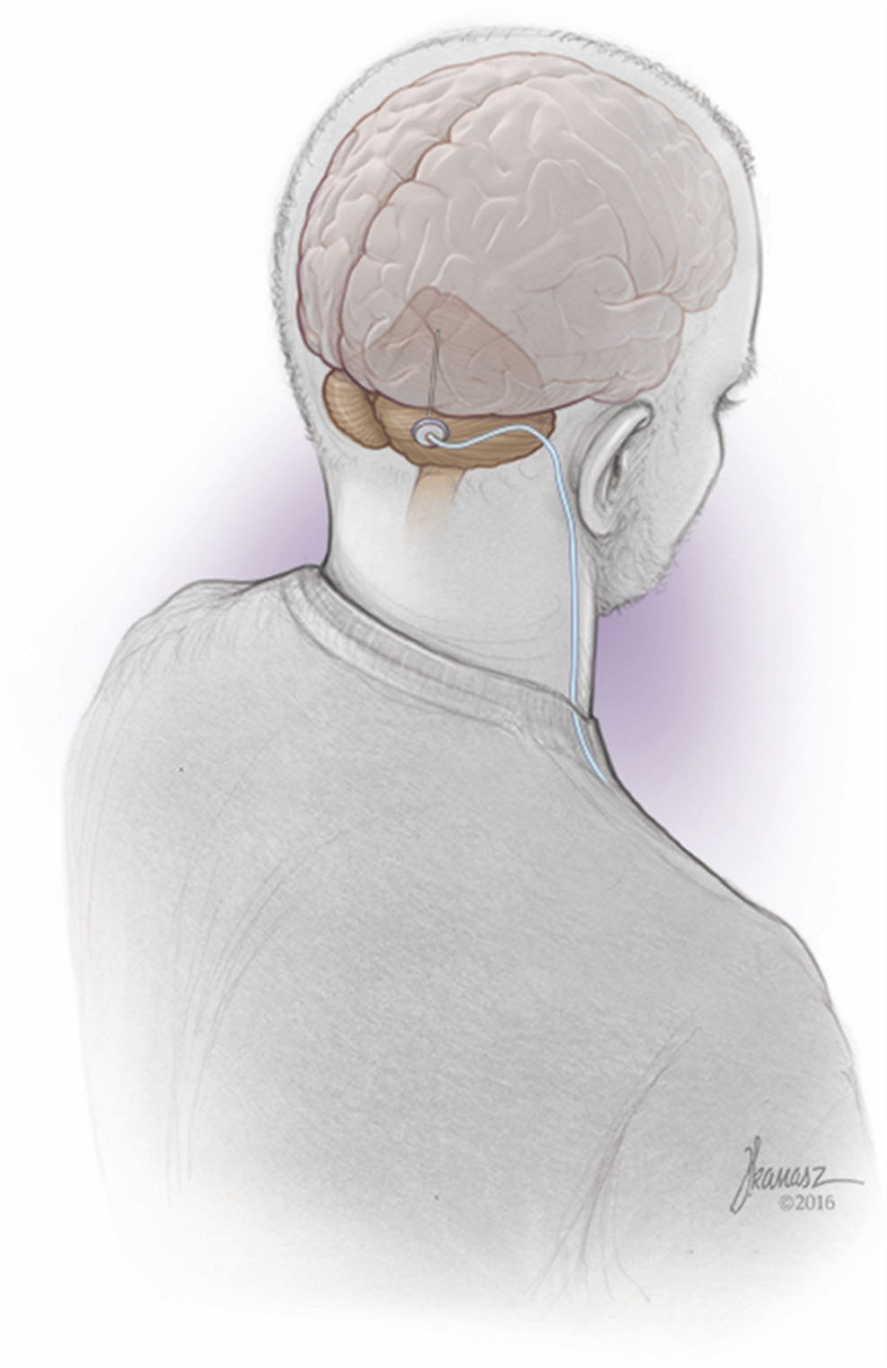
Deep brain stimulation of the human cerebellum. Cartoon drawing illustrating the general approach of deep brain stimulation targeting the dentate (lateral) nucleus in human

While pre-clinical studies in animal models have shown that cerebellar stimulation may be beneficial in the treatment of several diseases, including dystonia and epilepsy, its promise as a therapy for stroke is of particular interest due to the need for better treatments. Stroke is a devastating neurological event that disrupts brain function and causes neuronal death. Most stroke survivors suffer long-term deficits that range from motor and/or sensory dysfunction to speech or memory loss, depending on infarct site and injury severity. Post-stroke motor disability presents a substantial burden to the population, both in terms of individual quality of life and in the social and economic resources required to care for these patients. Current treatment for patients with motor sequelae is largely limited to physical therapy; however, many patients retain long-term disabling deficits despite best efforts. As such, there has been substantial interest in investigating the use of electrical or magnetic stimulation of the cerebral cortex to promote post-stroke functional recovery. Unfortunately, the efficacy of such approaches has, thus far, been variable or limited [114]. To this end, our group was the first to propose, research, test, and translate a novel neuromodulatory stimulation approach targeting the ascending dentatothalamocortical (DTC) pathway for post-stroke motor rehabilitation. This approach involves stimulation of the cerebellar dentate nucleus, the origin of the DTC pathway, in order to enhance activity along this natural excitatory fiber tract and augment thalamocortical interactions across multiple prefrontal, frontal, and parietal cortical regions (Fig. 3). We proposed stimulation of the DTC as part of a neuromodulation-based rehabilitation strategy for several reasons. First, single pulse stimulation of the dentate nucleus had been previously shown to modulate cerebral cortical excitability [115–117]. We extended those findings by showing that continuous stimulation of the dentate nucleus produces sustained, frequency-dependent modulation of cortical excitability in both naïve and post-stroke rodents [118, 119]. These results enabled our group to conclude that low-frequency beta band stimulation might optimally enhance cortical excitability and create an ideal environment for further promoting functional reorganization and recovery. Second, we hypothesized that chronic, exogenous activation of this excitatory pathway could reverse the crossed cerebellar diaschisis, and possibly even atrophic changes, that occur following contralateral cortical ischemia and contribute to loss of function [90, 94, 120].

Initial studies in our lab investigated the effect of chronic stimulation of the LCN (i.e., the homologue of the primate dentate nucleus) in rats with large, ischemic strokes of the middle cerebral artery, revealing significant enhancement of motor recovery with lower frequency stimulation [121]. Subsequent work examined whether stimulation combined with simultaneous motor training promotes recovery following small, cortical lesions [74, 122]. We found that dentate stimulation at 30 Hz produced significant gains in motor function compared to control animals and significantly enhanced the expression of synaptophysin in the perilesional cortex [74, 122]. Recently, a Stanford group has replicated the neurorestorative effect of DTC stimulation utilizing optogenetic stimulation instead of electrical stimulation in the mouse model [75].

In parallel with our optimization and behavioral work, we have sought to uncover the mechanisms through which DTC stimulation-induced recovery occurs. We have demonstrated that post-stroke stimulation is associated with significant synaptic changes in the perilesional cortex, including increased expression of PSD95, a marker of synaptogenesis, as well as an increase in the number of perilesional synapses [74]. DTC stimulation has also been associated with perilesional upregulation of markers of LTP, including CAMKII and the NMDA receptor [74]. In addition, stroked rats who received DTC stimulation with LCN leads have altered cortical motor maps, with increased representation of distal and proximal forelimb and decreased representation of the unaffected limb [74]. Furthermore, stimulation has recently been shown to be associated with increased neurogenesis in the perilesional cortex, as well as in the mediodorsal and ventrolateral thalamic relay nuclei, providing another mechanism through which the facilitative effects of stimulation may occur. Interestingly, stimulation was associated with greater glutamatergic and less GABAergic neurogenesis compared to control animals [123]. These data indicate that there are a number of associated microstructural, cellular, and potentially even neuroregenerative changes associated with DTC stimulation that may provide the mechanistic underpinnings of this neuromodulatory therapy.

Based on these promising pre-clinical data, a first-in-human phase I trial (Electrical Stimulation of the Dentate Nucleus Area (EDEN) for Improvement of Upper Extremity Hemiparesis Due to Ischemic Stroke: A Safety and Feasibility Study) has recently received approval and is actively enrolling (ClinicalTrials.gov Identifier: NCT02835443). This study will evaluate the safety and feasibility of dentate nucleus stimulation in conjunction with physical therapy in patients with moderate to severe upper-extremity hemiparesis following middle cerebral artery ischemia. Although significant differences exist between the formative rodent work and human application, initial results from the first implanted patient are promising and have inspired an extension of the original study timeline in order to examine not-yet-plateaued motor recovery. Additionally, this clinical trial holds great promise because it has been recently shown that cerebellar modulation via intermittent theta-burst stimulation improves gait and balance in stroke patients, possibly through neuroplasticity mechanisms (ClinicalTrials.gov Identifier: NCT03456362 [124]).

The dentate stimulation-associated microstructural and neural excitability changes are currently only correlative; future work will evaluate the causal mechanisms underlying its therapeutic effect. Deciphering whether the functional recovery achieved in pre-clinical studies is a result of stable reorganization of the cortex or whether the facilitatory effects of DTC stimulation will require continual stimulation to maintain benefits also needs to be examined. Future work in rodent and non-human primate models, as well as human studies, will also focus on optimization of stimulation timing and parameters. Finally, we postulate that stimulation of the DTC pathway may be beneficial in improving recovery from other types of cortical injury, including traumatic brain injury. Results of the first-in-human trial will soon be available and will drive future investigation.

## Cerebellar Stimulation in Humans: Clinical Applications

### Clinical Experience with Electrical Cerebellar Stimulation (T. Wichmann, M.R. DeLong)

The effects of chronic stimulation of the cerebellar cortex as a treatment for movement disorders were explored before, by a small number of investigators, in the setting of cerebral palsy (CP) with spasticity and dystonia (or “athetosis,” as it was commonly called [111, 125–144]) and epilepsy.

#### Cerebellar Stimulation for Treatment of Motor Dysfunction Associated with CP

The cerebellar cortex was a common target for treating the motor dysfunction(s) associated with CP [126, 143]. Alternatively, a transtentorial or suboccipital approach was used to implant deep wire electrodes into the anterior lobe of the cerebellum [133, 144], or into the brachia conjunctiva [140]. There was no consistency with regard to the stimulation conditions. For example, early studies tended to use chronic high frequency constant-current stimulation, while some of the later studies used chronic intermittent constant-voltage stimulation (e.g., 185 Hz, 210 μs, 15 min on, 2–6 h off [144]). Cerebellar stimulation was generally considered safe [135, 145, 146], but infections and equipment malfunctions were frequent [132, 142].

One of the pioneers in this field, Dr. Ross Davis, reported in 2000 that 600 CP patients had been treated with cerebellar stimulation in 18 different clinics [143]. The studies reported improvements in spasticity and athetosis [126, 127, 132, 143, 147], as well as gait [129] and respiration [137]. In some of these patients, the stimulation was also associated with psychologic improvements, such as reductions of anxiety or improved visuomotor functions [128, 140, 148]. While the results were not as impressive when studied in a double-blind fashion (compared to the earlier unblinded studies), the blinded studies, nevertheless, confirmed that about 2/3 to 3/4 of treated cases improved, and that at least 50% of the patients had a reduction of spasticity by “more than 20%” [138, 139, 141]. As has been the experience in patients undergoing basal ganglia DBS for dystonia, the effects were typically seen after a significant delay [131].

#### Cerebellar Stimulation for Tremor

There is emerging evidence that cerebellar circuit abnormalities and morphologic changes at the level of the cerebellum are important for the emergence of kinetic tremor, such as essential tremor ([36, 46–48, 149, 150]; see section by Kuo, Xie, and Louis). In fact, lesioning or DBS of the portion of the ventral motor thalamus (VIM) that receives cerebellar input has been a mainstay treatment for severe essential tremor for decades [151–154]. However, it is not clear whether cerebellar stimulation would have similarly beneficial effects, although studies of transcranial direct current stimulation of the cerebellar cortex have had promising results ([155]; see section by Manto and Oulad Ben Taib).

#### Cerebellar Stimulation for Epilepsy (See Section by S.V. Gornati, F.E. Hoebeek)

In the 1940s, it was known that electrical stimulation of cerebellum could control motor seizures [156]. Partially driven by the experimental findings that the output of the cerebellar cortex is inhibitory [157], several investigators explored how cerebellar cortical stimulation can be used to control seizures, which were known to be driven by cerebral hyperexcitability, by inducing inhibition. The efficacy of cerebellar cortical and cerebellar nuclei stimulation has been tested for therapeutic value in a wide variety of animal models in various species (mouse, rat, cat, and monkey) in which seizures had been evoked by genetic manipulations, chemical infusions, or neurostimulation approaches [158, 159]. Driven by the positive outcome of the experimental studies on cerebellar stimulation, the first epilepsy patients with refractory seizures were implanted with electrical stimulation paddles, which were positioned on the anterior cerebellar hemisphere, in the 1970s. These patients mostly received chronic low-frequency (10 Hz) cerebellar stimulation alternating between the left- and right-side, which markedly reduced the frequency of seizures for up to 3 years [111]. However, the first double-blind controlled studies of cerebellar stimulation in five patients with refractory seizures revealed no consistent effect of the stimulation on epileptogenic thalamo-cortical networks [160].

#### Cerebellar Stimulation for Parkinsonism and Dystonia

As mentioned above, cerebellar dysfunction is additionally implicated in some aspects of PD, specifically tremor, and dystonia [47, 49–52, 161–163], but little is known about the potential use of cerebellar surgery for treatment of Parkinsonism and dystonia. Indirect evidence suggests that DBS of targets in the basal ganglia or the pedunculopontine nucleus may work best for PD if they involve pathways that connect to the cerebellum [164, 165], suggesting that some of the therapeutic effects of these interventions might involve the cerebellum. Based on limited experience with low frequency repetitive transcranial magnetic stimulation (rTMS), cerebellar stimulation could help with proximal movements in some PD patients, although it may have adverse effects on fine motor skills [166]. It has also been speculated that cerebellar stimulation could be beneficial for treatment of levodopa-induced dyskinesias in PD patients [167–169] and motor symptoms in focal dystonia [170]. With regards to dystonia, cerebellar stimulation could help with some aspects of this disease [171], especially with posture, as recently shown in rodents by White and Sillitoe (2017 [63]; see section by Miterko, Beckinghausen, Sillitoe).

As our understanding of the pathophysiology of motor disease evolves, the use of functional surgical techniques and cerebellar stimulation may advance. Whether stimulation is delivered through invasive (e.g., DBS) or non-invasive means (e.g., tDCS, TMS), we are armed with new ways of targeting the motor circuit that prioritizes patient health and optimizes clinical outcome. In the remaining sections, we will outline current cerebellar stimulation paradigms targeted to treat a range of motor diseases in humans and consider their potential mechanims so that these therapies may be optimized.

## Deep Brain Stimulation in Humans

### DBS in Essential Tremor (S-H Kuo, T. Xie, E.D. Louis)

Essential tremor (ET) is a progressive disease characterized by bilateral kinetic, postural and intention tremors in the arms and hands, and with time, these tremors become larger in amplitude and slower in frequency [172]. A subset of ET patients will also have voice tremor and head (i.e., neck) tremor. DBS of the ventrointermediate (VIM) nucleus of the thalamus is one of the most effective surgical options for the treatment of ET. It can decrease tremor amplitude up to 50–80% [153] and be effective for over 7 years [173], making it the standard therapy for medication-refractory ET [174, 175]. However, decreased responsiveness to VIM DBS by select body parts (possibly due to the somatotopic organization of the VIM nucleus) increased tolerance to VIM stimulation due to compensation or disease progression [176, 177], and the development of adverse side effects, such as dysarthia and dysphagia [174], in patients, has driven efforts to optimize DBS targeting.

The VIM nucleus has specific features that likely support DBS efficacy and which would be ideal to preserve while selecting a new target for ET. For one, the VIM nucleus receives extensive cerebellar outflow fibers from the cerebellar nuclei [54]. Intra-operative recordings from the VIM nucleus showed that neurons fire rhythmically at the same frequency as the tremor [178]. Combined with the structural changes to the Purkinje cells [179, 180], possible Purkinje cell loss [181], Purkinje cell axonal alterations, and/or abnormal Purkinje cell synaptic organization [182, 183] reported in postmortem human ET tissue, the VIM neurons are likely entrained by abnormal cerebellar activity. Further supporting the idea that the cerebellum may be responsible for tremor generation, experiments conducted with animal models of harmaline-induced tremor have shown that enhanced coupling of the inferior olivary neurons can produce rhythmic discharges of the downstream cerebellum that drive tremor [184]. Additionally, this tremor can be effectively eliminated by VIM DBS in a frequency- and voltage-dependent manner [185]. Although there is no clear evidence of enhanced neuronal coupling in the inferior olives of ET patients [186], animal models of harmaline-induced tremor indicate that the abnormal physiology within the cerebello-thalamo-cortical loop can produce ET-like tremor.

The cerebello-thalamo-cortical loop is already implicated in the pathogenesis of ataxia, a clinical sign that is frequently comorbid with tremor. For example, ET patients often have subtle cerebellar ataxia manifested by difficulty in tandem gait [187], and a subset of ET patients will eventually develop frank ataxia [188]. However, with VIM DBS, effective tremor suppression can sometimes come with the price of worsening gait ataxia, a clinical observation that suggests that different neuronal coding mechanisms for tremor and ataxia lie within the same cerebello-thalamo-cortical loop [189]. Targeting the caudal zona incerta (cZi) and prelemniscal radiation (Rsprl) in posterior subthalamic area (PSA) seems to be as effective in tremor suppression as VIM DBS, but perhaps has better tolerance and fewer side effects of dysarthria, disequilibrium, or ataxia [190]. Like the VIM, the cZi and the Rsprl in PSA also receives innervations from the cerebellum and other brain regions such as the midbrain and basal ganglia. A long-term study with bilateral DBS placed across the cZi and VIM is required to truly compare the efficacy and adverse effects of these targets. In addition, the neuroanatomy and the mechanism as to why cZi or Rsprl in PSA might be superior targets to VIM for neuromodulation in tremor will need to be explored.

Since ET is a movement disorder of specific measurable variables—frequency, phase, and amplitude—DBS of different frequencies and currents in ET can serve as a model to probe how brain modulation could regulate real-time movements. For example, DBS may reduce tremor by regionally modulating neuronal activities in the VIM nucleus. In turn, this can lead to wide-spread activity changes in the brain network, including the cerebellum, as evidenced by differential cerebellar synaptic reorganization in ET cases with and without DBS [191, 192]. It remains to be elucidated whether direct stimulation of the cerebellum can equally suppress tremor or if clinical benefits are better when intervention is earlier or adaptive. It is possible that stimulation of the cerebello-thalamo-cortical loop earlier could modulate cerebellar activity and alter the structural and degenerative changes seen in the ET cerebellum. However, due to the progressive and wide-spread nature of ET, early stimulation may not be enough. Since tremor is a very unique movement disorder that can be characterized by phase and frequency, current stimulation paradigms (e.g., VIM DBS) can be optimized to these dynamics. For example, phase-specific VIM DBS can effectively modulate ET frequency and amplitude [193], paving the way for the development of adaptive DBS according to the tremor characteristics in *real time*. In fact, phase-specific VIM DBS has been shown to achieve tremor suppression with much less energy requirement [194]. Recently available directional leads would also help us to reduce energy consumption and prolong battery life, avoid side effects related to high DBS settings, and make more precise stimulation possible. Overall, future adaptive DBS would deliver stimulation on demand based on reliable biomarkers to guide automatic adjustments of stimulation, which would also lead to a better understanding of the brain circuitry of ET.

## Non-invasive Stimulation in Humans

### Cerebellar tDCS in Healthy Subjects and Diseased Patients (M. Manto, N. Oulad Ben Taib)

While DBS holds promise for correcting a range of abnormal motor behaviors in humans, it requires surgical interventions. However, the human cerebellum is easily accessible to non-invasive stimulation due to its anatomical location [195]. The technique of tDCS is a non-invasive method, which is gaining in popularity to probe and modulate cerebellar functions, both in healthy subjects and in cerebellar disorders [196]. The recently described anatomical communications between the cerebellum and basal ganglia extend the potential applications of tDCS to extra-pyramidal disorders, especially PD and dystonia [197]. Pathological modifications in the cerebellum circuitry, both neuropathological and functional, have been reported in PD and likely reflect a compensatory response to the hypofunction of the striato-thalamo-cortical pathway [198–200].

Transcranial DCS consists of the administration of a low-intensity current (0.5–2.5 mAmp) over the scalp with sponge electrodes. One electrode (cathode or anode) is applied over the cerebellum on the back of the skull, with a reference electrode either on the skull (in particular: over the motor cortex, prefrontal cortex, or over the buccinator muscle) or on the shoulder. Cerebellar tDCS modifies the excitability of the cerebellar cortex with minor side effects (mainly burning or itching sensation). Polarity of the electrodes dictates the effects on the cerebellum [195]. Anodal tDCS excites the cerebellar cortex, whereas cathodal tDCS exerts an inhibitory effect. Interestingly, the technique allows the application of a sham current. Modeling studies provide strong support for a direct effect of tDCS upon the cerebellar circuitry and for its remarkable containment of the current [197].

Furthermore, studies on the effects of cerebellar tDCS on healthy patients have revealed which circuits and functions can be modulated. Similar to DBS, tDCS impacts the cerebellum, the thalamus, the basal ganglia, and the cortex, as measured by the cerebellar brain inhibition (CBI), EEG, and behavioral indices [201–204]. For example, tDCS has been shown to modulate CBI in healthy subjects [205]. However, there is no consensus regarding the impact of cerebellar tDCS on CBI. Some authors have found a reduction of CBI following anodal stimulation of the cerebellum [206]. Possible explanations are that there is a direct effect upon the inhibitory interneurons of the cerebellar cortex or there is an effect of the cerebello-thalamo-projections upon the inhibitory interneurons of M1. Other evidence of a physiological effect of cerebellar tDCS upon brain circuitry include (1) a lateralized synchronization over the sensorimotor area in the gamma band and (2) an increase of the network segregation in sensori-motor rhythms with a greater communication between left-right hemispheres in the gamma band, by anodal tDCS [207]. The plastic modifications induced by cerebellar tDCS are particularly relevant given the numerous forms of plasticity encountered in the cerebellar circuitry [197]. In terms of behavior, cerebellar tDCS improves postural control following perturbations induced by Achilles tendon vibration and influences also the perception of pain [203, 208].

Given the connections affected by cerebellar tDCS, this technology holds promise for treating various motor diseases. A recent systematic review and meta-analysis supports this by providing evidence for a positive effect of non-invasive brain stimulation on motor symptoms [209]. Particularly by modulating the denato-cerebello-thalamic pathway and the activities of the prefrontal, parietal, and temporal lobes, the striatum, and subthalamic nucleus [53], movement disorders such as essential tremor, ataxia, Parkinson’s disease, and dystonia can be managed.

#### Transcranial DCS for Tremor

Since tremor can be associated with cerebellar pathology, especially at the level of the cerebellar cortex [210], and comorbid with ataxia, tDCS was targeted to the cerebellum and its downstream synaptic partner, the dorsolateral prefrontal cortex. Whereas the first randomized, double-blind, cross-over study with bilateral cathodal cerebellar stimulation showed no effect on essential tremor [155], the second study in which anodal tDCS was applied over the dorsolateral prefrontal cortex showed an improvement in ADL (i.e., Activities of Daily Living) scores and TETRAS (i.e., The Essential Tremor Rating Assessment Scale) scores [211]. Furthermore, tDCS over the cerebellum immediately followed by tDCS over the contralateral motor cortex reduces the amplitude of postural tremor and action in tremor in SCA2 [212, 213].

#### Transcranial DCS for Ataxia (See Also D. Timmann, M.A. Nitsche)

Cerebellar tDCS improves ataxia by (1) reducing the amplitudes of long-latency stretch reflexes in cerebellar ataxias, without an effect upon short-latency stretch reflexes [214] and (2) reduces hypermetric movements and improves the abnormal timing of agonist-antagonist EMG bursts. This suggests that tDCS strengthens the inhibitory effect of Purkinje neurons upon cerebellar nuclei and that tDCS improves muscle function. Furthermore, two studies from Benussi et al. (1: single session, 2: 2 weeks’ administration; double-blind, randomized, sham-controlled study) have shown a symptomatic benefit on ataxia scores (SARA and ICARS) and quantified measurements such as the 8-m walking time and performance scores from the nine-hole peg test [215, 216]. In particular, anodal cerebellar tDCS exerts a favorable effect upon the SARA score, ICARS score, and nine-hole peg test (9HPT) testing. A 2-weeks’ treatment with anodal cerebellar tDCS improves cerebellar symptoms and restores CBI as compared to the sham condition. However, a confirmatory study on a large sample of cerebellar patients is currently missing.

#### Transcranial DCS for Other Movement Disorders

Cerebellar tDCS has been applied in basal ganglia disorders, in particular PD and dystonia (see also T. Popa, M. Hallett). Anodal tDCS applied during five consecutive days over the motor cortical areas and the cerebellum improves the levodopa-induced dyskinesias in PD [217]. Cerebellar anodal tDCS improves the kinematics of handwriting and circle drawing tasks in patients with writing dystonia [218]. However, the effects of cerebellar tDCS upon dystonia remain controversial [219]. Cerebellar tDCS is promising to promote the rehabilitation for language deficits, in particular aphasia following a stroke [220], but the optimal location of stimulation requires to be defined. Anodal tDCS of the right cerebellum coupled with behavioral therapy is more efficient than behavioral therapy alone to improve spelling and dictation [206]. Interestingly, the resting state functional connectivity MRI data show that improved spelling is associated with an increase in cerebello-cerebral network connectivity. Cathodal tDCS enhances verb generation without modifying verb naming in post-stroke aphasia [221]. Together, these preliminary results open the door for a tDCS-based symptomatic management of numerous motor disorders.

### Cerebellar tDCS and Motor Learning (D. Timmann, M.A. Nitsche)

Because of its easy application, low costs, and promising initial results, tDCS of the cerebellum has gained interest in recent years for the treatment of ataxia and other movement disorders [195]. While the mainstay of treatment for cerebellar ataxia is physical therapy, or motor training, accompanied by occupational and speech therapy, neuromodulatory interventions are highly desirable therapeutic supplements. Not only does training improve cerebellar dysfunction in patients [222, 223], non-invasive brain stimulation has been shown to induce and enhance plasticity, a physiological process relevant for learning and memory formation, and is therefore a likely candidate to enhance cerebellar-dependent learning processes [224–226]. Transcranial DCS likely induces neuroplasticity through modulating the excitability of the cerebellar cortex. In accordance, cathodal tDCS reduces CBI, whereas anodal tDCS leads to increased CBI, at least at low intensities of the conditioning cerebellar TMS pulse [205]. The excitability of the primary motor cortex is thereby tuned by CBI.

Ultimately, these changes to brain activity can mediate learning, as evidenced by initial findings in reach adaptation and eyeblink conditioning experiments, which show that tDCS can improve both cortical and cerebellar-dependent learning [227, 228]. For example, Galea et al. (2011) found that anodal tDCS resulted in faster visuomotor reach adaptation compared to sham stimulation in young and healthy subjects [229]. Herzfeld et al. (2014) showed that anodal cerebellar tDCS improved force field reach adaptation whereas cathodal tDCS disrupted this learning ability [230]. Similarly, locomotor adaptation has been found to improve with anodal cerebellar tDCS and decline with cathodal tDCS [231]. Furthermore, the acquisition of conditioned eyeblink responses was fostered using anodal tDCS, but deteriorated with cathodal tDCS [232]. These results do not only imply that cerebellar tDCS can improve learning, but deliver also relevant mechanistic information. Since anodal tDCS induces long-term potentiation (LTP)-like plasticity, and improved learning, the results provide further evidence against the long-standing view that long-term depression (LTD) at the parallel fiber-Purkinje cell synapse is the only and the essential kind of plasticity underlying learning in the cerebellar cortex [233]. In accordance, a recent study in mice found that anodal tDCS effects depend on LTP and the intrinsic plasticity of Purkinje cells in VOR habituation [234]. This has been further supported in recent years by Johansson et al. (2015) and Gutierres-Castellanos et al. (2017) [235, 236].

Despite the promising initial results of cerebellar anodal tDCS on motor learning, recent studies showed that at least some of these findings are difficult to replicate. Firstly, Hulst et al. (2017) found no effects of neither cerebellar cathodal nor anodal tDCS on force field reach adaptation in young controls, elderly controls, and in patients with cerebellar degeneration [237]. Maybe most importantly, Galea and colleagues (2017) were unable to reproduce their initial findings in visuomotor reach adaptation using a very similar set-up and paradigm [238]. They found positive effects of anodal tDCS only for adaptation of movements of the right index finger, but not of movement of a digitizing pen (as in the original study conducted by Galea et al. 2011) [229]. They were unable, however, to reproduce the respective positive finding in a second group of young and healthy subjects. Inconsistent findings have also been observed in eyeblink conditioning. Timmann and colleagues (2017) were unable to reproduce their initial strong tDCS effects in studies using the same conditioning set-up [239]. Thus, prior to clinical applications, one needs to understand the reasons for these inconsistent findings. One important factor may be that effect sizes are much smaller than expected based on the initial positive findings, because of a bias towards publishing positive but not negative, results [238]. Furthermore, directionality, and the amount of tDCS effects, critically depend on the orientation of the nerve fibers, and the highly convoluted cerebellar cortex may be a reason that it is difficult to predict tDCS effects in an individual subject [240]. To make things even more difficult, zebrin positive and zebrin negative zones of the cerebellar cortex appear to be involved in different forms of motor learning (e.g., VOR adaptation vs. eyeblink conditioning), and use different learning-related plasticity mechanisms, that is LTP in zebrin positive zones, and LTD (and other mechanisms to suppress simple spike firing in Purkinje cells) in zebrin negative zones [241]. Thus, for cerebellar tDCS, it might be necessary to shape stimulation protocols to allow targeted and efficient intervention in future studies, including clinical applications.

To this aim, it may be helpful to develop predictors of tDCS efficacy. Here, sensitivity for CBI might be a promising candidate. Similar to the effects of tDCS on the primary motor cortex, which correlate with the sensitivity to TMS effects [242, 243], there may be a relationship between CBI and tDCS effects at least for certain motor learning tasks. Furthermore, systematic optimization of stimulation protocols, regarding stimulation intensity, duration, repetition rate, targeting, electrode arrangement, and computational modeling based on individual MRI scans to optimize stimulation protocols at the level of the individual might be helpful to increase efficacy of the intervention [243, 244]. For improving the understanding of the mechanisms of action of tDCS, and thus shape stimulation protocols on a physiology-based foundation, animal experiments are needed to comprehend tDCS effects on the level of different cerebellar layers, cell types including inhibitory interneurons, zebrin positive and negative zones, and the cerebellar nuclei. Finally, cerebellar tDCS effects likely depend on disease stage and ataxia type in patients with cerebellar degeneration; thus, individual adaptation of stimulation protocols due to the physiological and structural state of the cerebellum might be required. These multi-level activities are needed to systematically explore the utility of this intervention tool beyond small-sized pilot studies.

### Cerebellar Non-invasive Stimulation in Human Dystonia (T. Popa, M. Hallett)

Non-invasive brain stimulation studies for dystonia are scarce and an assessment of their efficacy is limited to dystonia types in which it is possible to have EMG recordings uncontaminated by muscle contractions; that is, focal/segmental dystonia and dystonic contractions in the setting of levodopa-induced dyskinesia. Like cathodal tDCS for ataxia, CBI values are decreased in eight subjects with focal hand dystonia [245]. This suggests that tDCS can similarly modulate cortical activity in dystonia.

Neuroplasticity can also be achieved by the non-invasive brain stimulation methods of intermittent TBS and rTMS. In healthy subjects, rTMS and tDCS can bidirectionally change the cerebellar cortex output for at least 30 min: 1 Hz rTMS, continuous theta burst stimulation (cTBS), or cathodal tDCS decreases CBI, while intermittent TBS (iTBS) and anodal tDCS strengthen it [205, 246]. When similar types of stimulation are applied prior to paired associative stimulation (PAS) with a 25-ms interval, which is a protocol to induce long-term potentiation-like plasticity in M1, PAS can be bidirectionally modulated: cTBS_cerebellum_ and cathodal tDCS_cerebellum_ lead to significant enhancement of PAS-induced M1 plastic effect above the ShamTBS_cerebellum_+PAS_M1_ level, while iTBS_cerebellum_ and anodal tDCS_cerebellum_ lead to its abolition [247, 248]. Interestingly, the enhancement of M1 excitability in the target muscle of healthy volunteers with median nerve stimulation, i.e., APB, following cTBS_cerebellum_+PAS_M1_ is accompanied by a non-specific excitability increase in an ulnar muscle, i.e., ADM [248]—a pattern of increased plastic response and loss of cortical map specificity similar to that described in focal dystonia explored with PAS_M1_ alone [249]. When this combined TBS_cerebellum_+PAS_M1_ paradigm was explored in patients with writer’s cramp, cerebellar cortex excitation and inhibition were both ineffective in modulating PAS-induced plasticity, suggesting a functional disconnection [250]. When this paradigm was explored in patients with cervical dystonia, cerebellar cortex excitation and inhibition induced the exact opposite modulatory effect on PAS-induced plasticity—a pattern observed also in healthy controls voluntarily maintaining a turned head or maintaining the head straight and having the sternocleidomastoid muscle vibrated [251]. This discrepancy suggests that the apparently common alterations in cortical excitability, sensory processing, susceptibility to undergo plastic changes, and wide-scale cortico-subcortical interactions do not have the same pathophysiology in different types of dystonia.

This conclusion emerges also from the several attempts made to use non-invasive stimulation of the cerebellum as therapy for focal dystonia. All trials addressing cervical dystonia obtained clinically positive, albeit modest, outcomes, while the trials addressing focal hand dystonia did not. A study using ten consecutive days of sham-controlled cTBS (600 pulses) delivered bilaterally over the posterior cerebellum of 20 patients with cervical dystonia led to a small (15%) improvement of the Toronto Western Spasmodic Torticollis Rating Scale (TWSTRS) and the recovery of the motor map responsiveness measured as a reduction of the heterotopic PAS_M1_ potentiation, i.e., only APB and not FDI excitability was responsive to PAS post-intervention [170]. In this study, no changes were found in Burke-Fahn-Marsden Dystonia Rating Scale, cortical silent period, intracortical inhibition/facilitation, or cerebellar-brain inhibition. The changes were found significant immediately after the 10 therapeutic sessions, but not at the 2- or 4-week follow-up post-intervention. Another study using an identical sham-controlled design, but with iTBS_cerebellum_, in 16 patients found a small but significant improvement in the severity and quality of life scores, but no changes in the cortical neurophysiological parameters [252]. While the cTBS_cerebellum_ study normalizing the exaggerated PAS_M1_ effect is in line with the reversed modulation finding [251], the iTBS_cerebellum_ study can appear counterintuitive. Both studies need further confirmation on larger cohorts. However, if the results of both studies are reproduced, it might suggest that any perturbation of the cerebellar cortex might be beneficial for cervical dystonia. A single-case, proof-of-concept study combined botulinum toxin with anodal tDCS in a cervical dystonia patient, applying the stimulation for 30 min, twice a week, over the right cerebellum (5 sessions), left cerebellum (5 sessions), and right M1+left cerebellum (10 sessions), switching the stimulation site when patient reported no benefit for two consecutive sessions [253]. The authors reported a 39% improvement in the TWSTRS (i.e., Toronto Western Spasmodic Torticollis Rating Scale) score and a 40% improvement in the quality of life questionnaires from one toxin injection to the other (12 weeks, 20 mixed-site stimulation sessions) without any other neurophysiological change. Another study reported that a single-session of cTBS over the right cerebellum paradoxically normalized the abnormal eyeblink classical conditioning in 10 patients with cervical dystonia [171]. This was opposite to the degradation of eyeblink conditioning observed in healthy subjects [254].

None of four studies using non-invasive cerebellar stimulation as therapy in focal hand dystonia found any significant clinical effect or a correlation between the neurophysiological parameters and the arm kinematics [218, 219, 255, 256]. This absence of acute clinical effects is not surprising especially after only a single session of cerebellar stimulation [257]. A common feature of deep brain stimulation of the globus pallidus, an emerging efficient treatment for certain types of dystonia [258], is that it often takes weeks to months for the alleviation of symptoms to occur [259, 260]. This is in stark contrast to other movement disorders like PD, which instantly and reliably benefits from either DBS [261] or a few sessions of rTMS [169, 262]. One possible explanation for this phenomenon is that dystonia is a network and/or plasticity disorder [263], and the delay represents the time necessary for the plastic changes to spread throughout the concerned networks. What is surprising is to have other types of dystonia respond acutely with clinical improvements to any kind of stimulation [170]. This behooves us to carefully consider generalizations of neurophysiological observations from one form of dystonia to another, and to not discount the idea that similar abnormalities (like an impaired CBI or exaggerated plastic response to PAS_M1_) might stem from different causes.

No explorations of the cerebellar output were attempted with non-invasive brain stimulation in other forms of dystonia. This leaves a big gap in our knowledge of the dystonic syndromes still to be characterized from an electrophysiologic standpoint. There is also an acute need of confirmation studies, especially regarding the clinical effects of cerebellar stimulation on human disease.

### Limitations of Non-invasive Stimulation for Therapeutic Use (M. Manto, N. Oulad Ben Taib)

Although studies have indeed shown that cerebellar tDCS is a promising treatment for a range of motor diseases, clear improvements are required, some of which have already been elucidated. Besides the need for larger sample sizes and confirmation studies, there is also a need to clarify what the ideal stimulation parameters are and what the patient pre-requisites are for benefitting from stimulation. For instance, in terms of elucidating the ideal stimulation parameters, a consensus must be reached on identifying the montage that needs to be used, defining the intensity and polarity of the current delivered, and optimizing intervals between sessions and the number of sessions. Furthermore, the type of disease, the duration of the disease, and concurrent treatments (e.g., motor training, see ref [264, 265], or pharmacotherapy, see ref [196]) might interfere with stimulation efficacy [266]. Therefore, large randomized controlled studies are needed to establish the efficacy in addition to a careful phenotypic characterization of cerebellar disorders, given their high heterogeneity. It is possible that some patients will benefit most off-line rather than on-line to non-invasive treatments or that efficacy is increased with patients having a greater cerebellar reserve. Efforts to assess on-line versus off-line effects and better quantify cerebellar atrophy are necessary. So far, it is hypothesized that (1) on-line benefits to stimulation might be the result of directly modulating Purkinje neuron activity, whereas off-line benefits may be the result from long-lasting changes to the activity of Golgi cells [267, 268], and (2) severe cerebellar atrophy with a major loss of neurons above a threshold is unlikely to respond well to non-invasive stimulation. Stimulating patients earlier, stimulating multiple sites, or delivering distinct modes of stimulation [265] are being considered, but have not been validated as viable alternative approaches, yet.

### Advantages of Non-invasive Stimulation for Therapeutic Use (L.V. Bradnam, A. McCambridge)

Non-invasive stimulation techniques—tDCS, rTMS, TBS—as mentioned above, are relatively simplistic, portable, are tolerated by patients (i.e., painless and has minimal side effects, see ref [269, 270]), and are low cost. In addition, these techniques require minimal training or supervision, making the delivery of semi-supervised home-based neuromodulation feasible [271], induce meaningful clinical effects from repeated sessions alone, and are highly amenable to experimentation, in both the basic and clinical research settings. That is why tDCS, for example, has been a primary research tool to study the motor system, non-motor processes such as cognitive and verbal functions, and overall cerebellar functions in both health and disease over the past decade. Although the exact neurophysiological mechanisms and resultant behavioral effects of ctDCS are not yet fully understood, we are making strides towards this through combining anodal stimulation paradigms with slice electrophysiology, eyeblink conditioning, and paired association studies to better understand cerebellar function, brain connectivity, and behavioral outcomes.

The proposed mechanisms underlying anodal ctDCS-induced neuromodulation are derived from direct current findings in animal slices and primary motor cortex (M1) tDCS in humans [272, 273]. Current research suggests anodal tDCS induces a subthreshold, polarity-dependent membrane polarization that induces neural plasticity via N-methyl-D-aspartate, gamma-aminobutyric acid, brain-derived neurotrophic factor, and calcium-dependent mechanisms [76, 274–276]. The neural circuitry underlying anodal ctDCS-induced effects on motor and non-motor function is not yet known, but is thought to involve the modulation of the cerebellar-thalamo-cortical route (see review [195]). Anodal ctDCS may facilitate cerebellar excitability by enhancing the inhibitory activity of Purkinje cells onto the deep cerebellar nuclei, thereby exerting less facilitatory drive to contralateral thalamic nuclei and the cerebral cortex [195]. The cerebellar-thalamo-cortical projections can be investigated in humans using TMS. The dual-coil TMS technique, termed CBI, delivers a conditioning TMS pulse to the cerebellum, followed by a test pulse to M1 to infer inhibition [201]. Studies have found that anodal ctDCS can influence activity in the cerebellar-thalamo-cortical pathway of healthy subjects and patient populations using dual coil TMS [205, 206, 215, 218].

One key study by Galea and colleagues (2009) extended earlier experimental findings [277] by also revealing polarity-dependent modulation of cerebellar excitability after ctDCS [205]. Cathodal ctDCS suppressed CBI in healthy adults, while anodal ctDCS allowed for CBI to be expressed with lower intensities of the conditioning pulse [205]. Increased inhibitory drive from the cerebellum was also noted in patients with cerebellar ataxia who underwent multiple ctDCS sessions [215]. However, the opposite result was reported by others, whereby anodal ctDCS suppressed cerebellar brain inhibition in healthy [206] and focal hand dystonia [218] participants.

Another method that infers cerebellar function of ctDCS in humans is delayed eyeblink conditioning. Based on findings in animals and support from neuroimaging and patient evidence, the cerebellum plays a key role in the acquisition, timing, and retention of conditioned eyeblink responses [278]. In this method, a reflexive eyeblink is acquired in response to a given stimuli (e.g., air puff) and repeatedly paired with a conditioning stimulus (e.g., loud tone). In comparison to sham ctDCS, the acquisition and retention of conditioned eyeblink responses after the conditioning stimulus alone was enhanced following anodal and reduced following cathodal stimulation [232]. Unfortunately, the same group were unable to replicate these findings using either a cephalic or extracephalic electrode montage [239], highlighting the poor understanding of the optimal stimulation parameters for ctDCS and issues with the replicability and reliability of ctDCS findings.

Another neurophysiological technique shown to be partially cerebellar-dependent is paired associative stimulation [247]. This technique involves repetitively pairing peripheral nerve stimuli and M1 TMS at distinct inter-stimulus intervals to induce LTP-like effects [279]. Anodal ctDCS blocked the induction of LTP, therefore indicating that human associative plasticity is influenced by the cerebellum [247]. Computational models show, as expected, that the cerebellum is the primary structure stimulated during ctDCS [280]. Yet further investigation of the after-effect of ctDCS on the cerebello-thalamo-cortical pathway and whole brain activity using various other methods such as neuroimaging is required.

Behavioral studies of anodal ctDCS commonly deliver stimulation concurrently with motor training. This idea is based on the hypothesis that increased cerebellar excitability induced by anodal ctDCS will facilitate motor performance, and concurrent training will enhance the functional specificity of tDCS to the neural circuits involved [281]. Several studies have found that anodal ctDCS can enhance the acquisition and/or consolidation of simple motor tasks by reducing movement errors [282, 283]. Interestingly, when performing a force field reaching task, anodal ctDCS increased the ability to learn from errors, plus form, and retain motor memory [230]. In contrast, there was no effect of anodal ctDCS on similar motor tasks in patients with cerebellar degeneration [237, 284]. This may indicate the importance of an anatomically functional cerebellum to mediate anodal ctDCS effects. The latter idea is supported by findings of performance improvements following anodal ctDCS in other small patient studies. For instance, anodal ctDCS improved dyskinesia scores in PD [217], and timing of agonist commands and tremor in cerebellar ataxia [212, 215, 216].

Lastly, the cerebellum is known to have a broad influence and makes a strong contribution to non-motor domains such as cognition [285]. Thus, it would be expected that anodal ctDCS would also modulate non-motor processes. Several preliminary studies have reported positive effects of anodal ctDCS on verbal fluency [286] and pain perception [287] in healthy subjects and cognitive symptoms in Parkinson’s patients [217]. While other studies have observed no effect on cognitive learning [288] or memory [289], for example. A meta-analysis of cognition studies found cognitive processes were influenced by anodal ctDCS but to a lesser extent than motor-related effects [290]. Whether this disparity is due to the sensitivity of assessments or a weaker influence of the cerebellum on cognitive processes is still uncertain.

Overall, emerging evidence provides some support for anodal ctDCS as a neuromodulatory tool for motor and non-motor functions. But the lack of replication is a significant concern that must be addressed. As recommended for tDCS research in general, the factors that underlie inter-individual variability must first be determined, as substantial variability will pose an additional challenge when exploring tDCS-induced effects in inherently heterogeneous patient groups. Nevertheless, there are several practical advantages of ctDCS in comparison to other therapeutic techniques and brain stimulation protocols that make tDCS a promising tool. And with better elucidation of its mechanisms of action and the neuronal circuitry that mediate reliable neurophysiological and behavioral effects, large clinical trials of the efficacy of ctDCS can implemented.

## Mechanism(s) of Cerebellar Stimulation

### Potential Mechanism(s) of Action of Neuromodulation: Lessons Learned from Stimulating the Basal Ganglia (T. Wichmann, M.R. DeLong)

Successful development of neuromodulation of cerebellar output (alone or in combination with modulation of basal ganglia output) relies on a better understanding of the functional changes that occur in the cerebellum and its output pathways in movement disorders and a better understanding of the mechanism(s) by which neuromodulation, such as DBS, works. Studies of the mechanisms of action of DBS have mostly focused on basal ganglia thalamocortical circuits in animal models and patients with PD. Although these studies have benefited greatly from our extensive knowledge of the pathophysiology of PD [291], a clear therapeutic principle has not yet emerged, complicating the search for more effective therapy targets or treatment strategies for PD (including “on demand,” biomarker-based therapy) or other movement disorders.

Such studies have taught us several lessons, however. One is that the effects of electrical stimulation on brain network activity are complex mixtures of activation and inactivation effects that involve activity changes downstream and upstream from the stimulated brain area [292, 293]. Another lesson from studies of the effects of DBS is that stimulation of the same sensorimotor targets in the STN- and GPi-DBS are effective in treating a variety of hypo- *and* hyperkinetic movement disorders. It, thus, appears that these interventions do not counteract specific aspects of the pathophysiology of the individual movement disorders, but non-specifically replace and block abnormal activity in the basal ganglia networks that target the relatively intact downstream portions of the motor circuitry [294–296].

Of course, the physiologic effects of electrical stimulation of the basal ganglia may be very different from those that would occur with stimulation of the cerebellum. However, if the interactions between the cerebellum and the basal ganglia thalamocortical circuits are of any significance in movement disorders, it may be most effective to concentrate on the goal of stimulating cerebellar efferents that either directly or indirectly influence basal ganglia function. While the benefits resulting from chronic stimulation of the cerebellar cortex were significant [67, 131, 297–306], it may be easiest to focus invasive stimulation approaches (such as DBS) on the deep cerebellar nuclei (or on the pathways emanating from them), given the substantial topographic spread of motor representations along the cerebellar cortex. To optimize this approach, a good understanding of the functional connectivity of specific deep cerebellar nuclei would be needed. The functional compartmentalization of the dentate nucleus into motor and non-motor regions [93] indicates that the precise localization of stimulation electrodes within specific functional regions of these nuclei could be of utmost importance for the development of such therapies.

Limited electrophysiologic measurements made during some of these interventions resulted in the belief that stimulation of the cerebellum has inhibitory effects on thalamic areas that receive cerebellar inputs, and on motor cortical areas [67, 297–299, 301, 302]. The effects were robust enough that significant inhibitory effects on somatosensory-evoked potentials were considered to be a prognostically favorable sign in patients with spasticity [300]. More recent studies have suggested that, at least under certain conditions, cerebellar output to the thalamus and cortex may instead have excitatory effects (see other sections in this paper, and studies by [303–305]). The method of cerebellar stimulation may explain some of the discrepancies in the literature. For instance, while electrical stimulation of the cerebellar cortex may have activated Purkinje cell output to the deep cerebellar nuclei (and may, thus, have had produced inhibitory effects in brain areas receiving cerebellar output), recent studies have suggested that at least some forms of TMS of the cerebellum (e.g., continuous theta-burst stimulation) lead to reduced cerebellar activity ([306]; see section by Popa and Hallett).

As for basal ganglia stimulation, it may not specifically matter whether cerebellar output is increased or decreased, as long as abnormal output from the basal ganglia (or cerebellum) is prevented from reaching the cerebral cortex. With regard to the effects of cerebellar stimulation on spasticity and other movement disorders, it is interesting that the effects are often delayed [131], just as they are in dystonic patients who are treated with pallidal interventions, suggesting that the stimulation may not only act to acutely alter synaptic transmission in brain areas downstream from the stimulation, but may lead to prominent subacute or chronic plastic changes in thalamus or cortex. In the following four sections, we consider these issues further, but instead from the perspective of the basic cellular level connectivity of the cerebellar cortex and nuclei.

### Electrical Stimulation of the Cerebellar Cortex (D.H. Heck)

Electrical stimulation of the cerebellar cortex provided among the first insights into basic principles of cerebellar network function in vivo [307–310] and in vitro [311, 312] was used in elucidating key principles of cerebellar cortical interactions with the cerebellar nuclei [10], allowed the generation of early cerebellar motor maps (particularly oculomotor maps) [313–318], and promised therapeutic potential [142, 319, 320]. However, a key limitation of electrical brain stimulation exists: electrical stimulation will not primarily activate the cell bodies of neurons surrounding the tip of the stimulation electrode, but instead activates predominantly axons [321–323]. This, of course, includes axons passing through the target area and will result in a mix of antidromic and orthodromic activation of fibers of passage as well as fibers that do originate in the target area [324–326]. This is particularly problematic when small nuclei embedded in white matter, such as the cerebellar nuclei, are the target. But this problem is also relevant for cerebellar cortical stimulation if the electrode tip is placed at a depth where stimulation could cause the antidromic activation of mossy fiber axons. Stimulation of the surface of the cerebellar cortex minimizes the risk of activating mossy fibers and will instead mostly activate parallel fibers which will in turn provide excitatory input to Purkinje cells and molecular layer interneurons [309].

In experiments where electrical stimuli were directly applied to the surface of the cerebellar cortex but also at various depths below the surface, John Eccles and a group of pioneering cerebellar electrophysiologists (1966) observed an excitatory response that propagated along the parallel fibers and was flanked on either side by inhibitory responses [309]. From these findings emerged the concept of the “beam” of activated parallel fibers as a geometric representation of a possible principle of neuronal computation in the cerebellar cortex [309, 327]. The “beam” concept emphasized the potential functional significance of the orthogonal arrangement of excitatory (parallel fiber) and inhibitory (stellate and basket cell) axonal projections, a unique characteristic of the cerebellar cortical network [19]. The combination of the unusual geometrical network architecture and the characteristic simple spike/complex spike waveforms that readily identified Purkinje cells [328] were likely responsible for the fact that most of the early electrophysiological investigations of cerebellar function focused on the cerebellar cortex, neglecting the role of the cerebellar nuclei. However, understanding cerebellar function requires understanding how the cerebellar cortex, hence Purkinje cell activity, modulates the activity of the cerebellar output neurons in the cerebellar nuclei. Electrical stimulation of the cerebellar cortex in vivo combined with in vitro experiments was used by Person and Raman (2011) to show that synchrony in Purkinje cell firing causes synchronized spike firing in cerebellar nuclear cells time-locked to that of the Purkinje cells [10].

Gordon Holmes’ studies of cerebellar deficits in WWI veterans (1917) had firmly established the cerebellum as a key player in the coordination of movements, including eye movements [329]. Later, electrical stimulation of the cerebellar cortex (mostly in cats) was employed to determine whether motor representation in the cerebellar cortex was topographically organized, or whether a cerebellar motor map existed. The results were rather complex. Cerebellar stimulation could elicit both simple movements and complex motor sequences, depending on stimulation site, stimulus amplitude, and frequency (e.g., [313, 314]). But, while results on body and extremity movements were quite variable, these experiments most prominently identified cerebellar cortical sites whose stimulation reliably elicited eye movements [315–317]. Those sites are now considered to jointly constitute the widely studied “oculomotor” cerebellum [330].

Most applications of electrical cerebellar cortical stimulation used single-site stimulation techniques and varied the temporal characteristics and amplitude of the stimulus applied to the site. If bipolar electrodes are used, the polarity can be switched to move the stimulus to the other pole, but stimulation is always at one site at a time. If multiple electrodes are used, it becomes possible to generate spatio-temporal activity patterns that allow the investigation of cerebellar network responses to dynamic events that cannot be studied with single site stimulation. Such a multi-electrode arrangement was, for example, used to demonstrate the ability of the cerebellar cortical parallel fiber system to transform sequential inputs to the granule cell layer into synchronous inputs to postsynaptic Purkinje cells [312, 331].

### Non-human Primates: Physiology, Lesion, and Stimulation of Cerebellar Nuclei (M. Tanaka)

Now, interest has shifted to modulating the activity of the cerebellar nuclei, instead of the cerebellar cortex, in hopes to increase efficiency and reduce the variability of outcomes of neurostimulation techniques [151]. The cerebellar nuclei have important properties and functions that make them a promising stimulation target. For one, the cerebellar nuclei guide a range of motor behaviors via its connectivity, activity, and computations. On the systems-level, the cerebellar nuclei outputs directly regulate movement signals in the brainstem and spinal cord, boost motor commands in the cerebral cortex via the thalamus, and modulate signals for adaptive learning through inhibitory projections to the inferior olive. Within the cerebellar nuclei themselves, they usually show a high baseline firing rate and exhibit transient activity during limb, hand, eye, and eyelid movements [332]. Additionally, a subset of neurons in the interposed and dentate nuclei also exhibit sustained, preparatory activity preceding movements [333], indicating the roles for the lateral cerebellum in motor planning [334]. Consistent with this, cerebellar lesions attenuate cortical readiness potentials [335], and the regional blood flow in the cerebellum correlates with the magnitude of contingent negative variation which predicts the occurrence of relevant events [336]. Recent studies in non-human primates demonstrated that neurons in the cerebellar dentate nucleus exhibited a gradual buildup of activity before self-initiated saccadic eye movements [337, 338], and that electrical stimulation applied to them facilitated self-timed, but not reactive, saccades [338]. Similar ramping neuronal activities were also found in the ventrolateral (VL) thalamus [339], and inactivation of the thalamus delayed self-timing [340], suggesting that the preparatory signals in the cerebellar nuclei might be sent through the thalamus to the cortex and regulate the timing of movement decisions. Irrespective of the length of the delay period, neurons in the dentate nucleus always started firing approximately a half-second before self-timed movements [338], while those in the striatum exhibited ramping activity throughout the delay period [341]. Since the neuronal correlates of trial-by-trial variation of self-timing emerged earlier in the cerebellum than in the striatum, the stochastic variation of self-timing might be primarily responsible for neuronal signals in the cerebellum [341].

Computationally, the cerebellum also plays a role in predicting sensory consequences of movements by calculating prediction errors that eventually alter subsequent movements. It has been well established that the cerebellum is essential for adaptive motor learning, which optimizes the force and timing of individual muscle contractions for accurate movements, and recent studies also suggest a role for the cerebellum in higher-order adaptive control of actions [342]. For example, it has been shown that the error-related cortical potentials during anti-saccades are reduced in subjects with focal cerebellar lesions [343] and that the magnitudes of the potentials correlate with the volume of Crus I and II in patients with cerebellar degeneration [344]. The other study used the stop-signal reaction time task and demonstrated causal relationships between cerebellar activity and error-related activation in the thalamus and the supplementary motor area, which in turn correlated with activation in the lateral prefrontal cortex during post-error slowing [345]. These results suggest that the cerebello-thalamo-cortical pathways may play roles in error detection and subsequent behavioral adjustment. In monkeys, neurons in the cerebellar dentate nucleus showed enhanced activity during both correct and erroneous anti-saccades [346]. Inactivation of them shortened the latency and deteriorated the accuracy of anti-saccades, while the proportion of error trials modestly increased [346]. During tasks requiring deliberate control, the cerebellum may predict error in advance of sensory feedback and may activate the frontal cortical network to alter behavioral strategy for subsequent movements [345], while the cortico-basal ganglia pathways execute proactive inhibition for the difficult tasks [347].

**Fig. 4:**
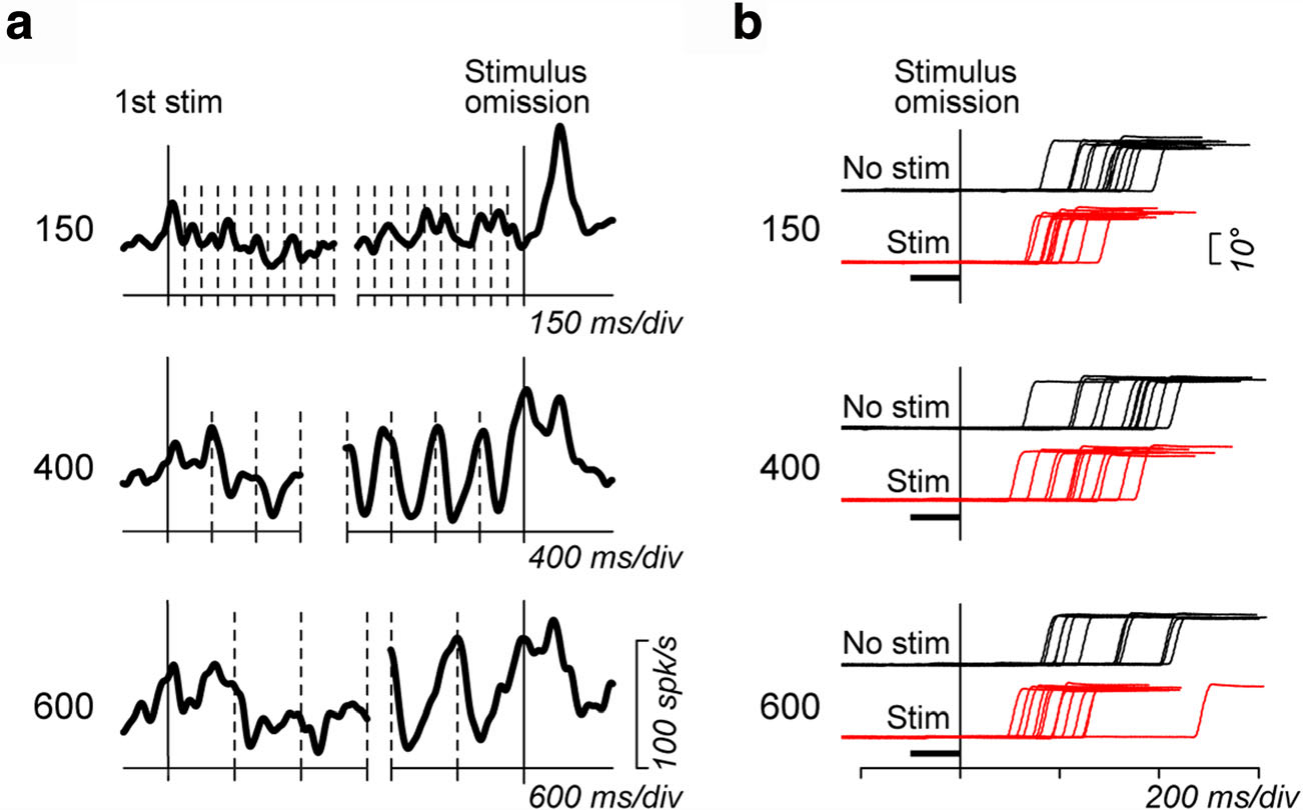
Electrical stimulation to the dentate nucleus advances sensory prediction. **a** Neurons in the dentate nucleus exhibited firing modulation when the monkey attempted to detect a single omission of periodic visual stimuli. **b** Electrical stimulation applied to the recording site shortened the reaction time for the stimulus omission. Adapted with permission from [350]

Besides motor control, many recent studies in humans show cerebellar nuclei involvement in non-motor cognitive functions [348]. Evolutionally, the lateral cerebellum is well developed in primates, and the associated dentate nucleus in humans comprises approximately 92% of total neurons in the cerebellar nuclei while this proportion is 26% in cats [334]. Anatomical data show that the ventral portion of the dentate nucleus provides signals to the association areas in the cerebral cortex through the thalamus, suggesting that these pathways are crucial for higher-order cognitive functions [91]. To date, only a few studies have explored the neuronal correlates of non-motor functions in the lateral cerebellum in experimental animals. In cats, Purkinje cells in the cerebellar Crus I have been shown to exhibit sustained activity for a moving object even when the object was temporarily removed, indicating that these neurons represent an internal model of external objects [349]. In monkeys, neurons in the dentate nucleus have been shown to exhibit a gradual increase of sensory gain when the animals attempt to detect a single omission of isochronously presented visual stimulus (Fig. 4a; [350, 351]). For these neurons, the inter-stimulus interval appears to be represented by the magnitude of firing modulation for each stimulus, and the time course of neuronal activity during each inter-stimulus interval accurately predicts timing of the next stimulus. Inactivation of these neurons delayed [351], and electrical stimulation promoted (Fig. 4b; [350]) the detection of stimulus omission, suggesting that they may provide temporal prediction of stimulus occurrence, which is needed to compute prediction error for the absence of regular stimuli [352]. Similar to the time course of neuronal activity in the cerebellar nuclei, temporally specific periodic signals predicting event timing have also been reported in the beta-band coherence of neuromagnetic activity between the cerebellum and the cerebral cortex when listening to an auditory beat [353], suggesting that such signals may provide a basis for the perception of rhythms. For temporal information processing, another line of evidence also suggests that the cerebellum might play a role in Bayesian inference of event timing [354, 355], although the underlying neuronal mechanism needs to be clarified in future studies in experimental animals.

### Cerebellar Compartmentalization: the Likely Involvement of Zones and Stripes in Neuromodulation (L.N. Miterko, J. Beckinghausen, R.V. Sillitoe)

Evidently, the largest hurdle to overcome will be to solve the mechanism of action in DBS, for each circuit, in each disease. From a general perspective, the cellular, circuit, and network effects of DBS have been debated at length [294, 356], and there are several different aspects of a potential “cerebellar mechanism” that could be discussed. Above, we discuss the effects of modulating cerebellar cortical and cerebellar nuclei activity on circuit functioning and behavior. Here, we would like to consider a possible mechanism from the view of the normal internal organization of the cerebellum.

All aspects of cerebellar development, function, behavior, and in many cases disease are organized around a striking array of parasagittal stripe domains, or zones as they are often called [3, 24, 357, 358]. At the center of each stripe are the Purkinje cells, the sole output of the cerebellar cortex. Remarkably, more than 30 years ago, it was recognized that stimulating adjacent regions of the cerebellar cortex—or the stripes—resulted in different behavioral outcomes in cerebral palsy patients that showed symptoms of dystonia [140]. These neurosurgical data are supported by electrophysiology studies in non-human primates showing that the normal cerebellum controls co-contractions of agonist and antagonist muscle activity [359], as well as transsynaptic retrograde tracing of the muscles that reveal connections to Purkinje cell stripes in rats [360]. The key to this architecture is that the Purkinje cell stripes and their associated climbing fiber inputs operate as synchronous units [361, 362]. The synchronous activity is processed within the deep nuclei, but here the signals from Purkinje cells must converge [10]. Thus, what type of activity are we tapping into when we stimulate the cerebellar nuclei using DBS? There are likely retrograde effects within the cerebellar cortex itself, but it would be interesting to test whether the responses within the thalamus and other downstream targets operate according to the topography that originates from within the cerebellum. If this is true, one must reconsider the possibility that cerebellar DBS could have incredibly variant effects depending on which specific sets of cerebellar zonal modules are recruited. These effects could manifest at the levels of cells, molecules, and circuits, which all contribute to the cerebellar zonal map. The use of conditional mouse genetics combined with optogenetics and DREADD approaches lend themselves to testing these possibilities in different disease models. Furthermore, optogenetic stimulation, as used by Gornati, Hoebeek, Cheng, Wang, and Steinberg for epilepsy and stroke, can help us understand cerebellar involvement in disease and cerebellar plasticity during rehabilitation. We already know that cerebellar DBS [74] and optogenetics [75] may be both effective as therapies, in addition to can be adjusted to mimic the parameters of one another [363]. Closed-loop DBS [364] as well as closed-loop optogenetic [365] methods could be very effective at elucidating cellular biomarkers in disease. As the techniques become more integrated and sophisticated, one could consider approaches such as near-infrared light technology alone or combined with optogenetics [366]. The complexity of the zonal cerebellar circuitry as well as its wide-spread connectivity in motor and non-motor behavior necessitate an equal level of sophistication in examining the responses to cerebellar stimulation. It will be imperative that single-unit electrophysiology approaches are used to analyze the cell-specific details of neuronal responses downstream of stimulation, but also population-level responses will have to be collected using tools such as tetrodes, array electrodes, silicone probes, or even fiber photometry and deep tissue endoscopy.

### The Potential Molecular Mechanisms Underlying the Efficacy of Cerebellar Stimulation (L.N. Miterko, R.V. Sillitoe)

The various mechanisms by which neurostimulation elicits large-scale, behavioral responses range from inducing plasticity to modulating local and global neural network activity, as previously discussed by Bradnam, McCambridge, Wichmann, DeLong, Heck, Tanaka, and colleagues, and regardless of the paradigm (i.e., tDCS, electrical stimulation, lesioning, optogenetics). While changes in plasticity and local or systems-level electrophysiological properties depend on what is being activated (axons vs. somata) and the region-specific functions that are being capitalized on by neurostimulation, there are common molecular threads that underlie learning and neural communication. For example, learning is a process shared among neurons throughout different brain regions and involves an increase in the influx of intracellular Ca^2+^ so that receptors involved in learning (e.g., mGluR1) can be activated. In turn, the activation of learning receptors can induce ultrastructural changes in the neuron, especially at the synapse (e.g., greater spine density), to promote sustained activity. Also common among neurons is their ability to communicate with one another. The release of neurotransmitters is a critical feature of action potential propagation and can be specific to the behavior by facilitating communication between cells in particular neural circuits. For instance, dopamine and serotonin can regulate emotion and support feelings of addiction and reward. Furthermore, cholinergic signaling is commonly associated with satiating hunger and glutamatergic signaling is commonly associated with learning and memory. While the roles of neurotransmitters are dynamic, and can switch depending on age and circumstance, the fact that neurotransmitter classes are well-elucidated, and we are learning more and more about their contributions to health and disease, creates optimism that we can uncover the molecular mechanism(s) of neurostimulation. However, it is still not clear whether neurostimulation approaches simply piggy back on the endogenous molecular pathways or whether stimulation in fact hijacks one or more pathways to shape neural activity for behavioral improvement.

Efforts to elucidate the molecular mechanisms of neurostimulation approaches transcend methods and brain regions. Of much interest to scientists and clinicians alike is how deep brain stimulation works since this is an FDA-approved method that is already readily employed in humans. Early studies on the molecular mechanisms of PD entailed a combination of fast cyclic voltammetry (FSCV), microdialysis, high performance liquid chromatography electrochemical detection (HPLC-ECD), and functional imaging (PET) [367, 368]. Increased tyrosine hydroxylase (TH) expression accompanied increased dopamine in the striatum of Parkinsonian rats after STN stimulation [367, 368]. Furthermore, behavioral improvements were documented after STN stimulation increased dopamine metabolism [368, 369]. However, these results were not reproducible in humans [370], and even if they were, it would not explain why DBS works despite patient resistance to dopaminergic drugs. Fluctuating serotonin levels have recently been implicated in the after-effects of STN DBS due to data reporting that (1) cognitive and depressive symptoms develop after stimulation and (2) anti-Parkinsonian drugs reduce serotonin in the prefrontal cortex and hippocampus, and (3) selective serotonin reuptake inhibitors (SSRIs) such as fluoxetine enhance responsiveness to neurostimulation [371–373].

While measuring neurotransmitter concentration and imaging its release gave insight into possible molecular mechanisms of DBS, more sophisticated methods have been developed to analyze molecular and genetic changes that occur on the single-cell level. Forniceal DBS is one example where quantitative PCR, RNA-sequencing, bisulfite sequencing, and proteomics were performed to address how electrical stimulation improves learning, memory, and promotes neurogenesis, as previously found by and Hao, Shirvalker, and colleagues [374–376]. Through a combination of these molecular and stimulation techniques, Pohodich et al. (2018) found that forniceal DBS in mouse upregulates genes involved in cell survival, synaptic function, and neurogenesis, specifically through Jun signaling [374]. These data are particularly interesting since earlier stimulation studies support the role of DBS in promoting neurogenesis through findings that electrical fields can (1) selectively direct the migration of stem cells [377–379], (2) increase blood flow [380], and (3) modulate neural networks (see sections by Cooperrider, Manto and Oulad Ben Taib, Bradnam and McCambridge, Popa and Hallett, and Timmann and Nitsche).

Are the molecular insights we have gained from studying STN and forniceal DBS applicable to cerebellar stimulation? There is a breadth of evidence suggesting that there may be some similarities. Not only does stimulating the cerebellum result in increased plasticity (see section by Cooperider and colleagues), it has also been found to be associated with dopamine release [381], increased blood flow [382, 383], non-motor relief [384], and modulatory effects on global neural networks [202, 385, 386]. Despite these shared properties, it is likely that molecular profiles diverge, especially as the paradigm changes. For instance, different locations (e.g., dentate vs. interposed vs. fastigal nuclei), frequencies (e.g., low vs. high), and pathologies most likely elicit different molecular changes in order to achieve improvements in varied behaviors. Studies on the use of DBS for essential tremor highlight this point. While Bekar et al. (2008) found that DBS may non-synaptically activate adenosine A1 receptors, leading to a reduction in tremor activity, this neuromodulatory effect may be specific to this pathology and thalamic targeting [185].

Understanding the molecular underpinnings of neurostimulation will greatly advance the treatment of diseases originating from or involving the cerebellum. Expanding studies to simultaneously address the effects of chronically implanting electrodes into the cerebellum will also deepen our knowledge of how to best treat diseases. It is already known that metal electrodes can promote gliosis, scarring, and prolonged inflammation, but can chronic implantation have any benefit [387]? Electrical stimulation may counter some of the negative effects of implantation, such as it can modulate inflammatory responses [388], but can implantation itself be additive and positively alter gene expression? It will be interesting to discover what stimulation versus implantation does, in addition to resolving whether stimulation at different frequencies and locations have distinct benefits. Solving these challenging problems will promote the design and implementation of treatments and therapies for targeting the many disorders with cerebellar involvement.

## Consensus and Summary

We present 17 different sections reviewing data for what is currently known about cerebellar stimulation in humans, non-human primates, and rodent models. Based on studies of electrical stimulation in the normal brain and in disease (human and animal models), there is a general consensus that stimulating the cerebellum has profound effects on behavior. Still, from a therapeutic perspective, one has to ask (and perhaps re-ask) the most direct and fundamental questions: does cerebellar neurostimulation *actually* work? If yes, how does it work?

Through previous experiences with cerebellar stimulation, it seems evident that at least some patients were significantly helped by these efforts, and that chronic cerebellar stimulation can be done without undue risk (see sections by Wichmann and DeLong). Clearly, stimulating the cerebellar cortex has a powerful influence on the circuit, and the response conforms to the structural framework within the cerebellar layers as well as their interaction with the cerebellar nuclei (see section by Heck). Cerebellar nuclei stimulation has similarly convincing effects, particularly in the behavioral domain in which motor and non-motor responses are modulated (see section by Tanaka). However, does this mean that placing stimulating electrodes into a cerebellar region alters the activity within that region? This has been a challenging problem to address since most often the stimulating current creates enough noise that in vivo recording of the responses at the stimulation site are masked. Based on analysis of neuronal activity in mice that are genetically modified to exhibit dystonia, it is suggested that stimulation indeed could have local effects on the output properties of cerebellar neurons [63]. Although, is it enough just to modulate cerebellar activity? Might there be additional long-distance changes that occur after cerebellar stimulation? The result of cerebellar stimulation in stroke certainly argues that cerebellar stimulation can induce plasticity in regions as distant as the cerebral cortex (see section by Cooperrider and colleagues) and these changes may be dependent on specific molecular mechanisms (see section by Cheng and colleagues). Even with this apparent specificity, it is difficult to rule out the possibility that stimulation could induce several anterograde and retrograde effects, and they could be local or long-distance [389]. For DBS, this leads us back to the question that remains unanswered: what is the mechanism of DBS? The general concept of DBS is that high-frequency stimulation modulates erroneous neural activity and entrains it to a pattern that normalizes behavior. There are a number of possible mechanisms [390], but one perspective is that the pulses produce inhibitory neuronal effects on somata that are proximal to the location of the electrode. The inhibitory action could be the direct result of a depolarization block through a mechanism involving sodium channel inactivation and potassium current potentiation. However, DBS might also increase and regularize the output of the stimulated region by activating local axons—this is certainly an appealing hypothesis in cerebellar disorders since much of electrophysiological defects could stem from changes in the firing regularity of the cerebellar nuclear neurons. At the network level, the end result is that the entrainment overrides pathological oscillatory activity. Perhaps cerebellar long distance connectivity and oscillations are key features that support DBS-mediated motor improvements in ET (see section by Kuo and colleagues). Again, given the potential dependence of cerebellar function on neuronal synchrony, it could be that DBS ultimately serves to normalize activity within an existing internal framework that packages cerebellar circuits into distinct functional modules.

Is the purpose of cerebellar stimulation to correct cerebellar function itself, or is it more important that it improves its functional connectivity with the remainder of the brain? There is also a third possibility, which is that the most critical outcome of cerebellar stimulation is to enhance connectivity between other brain regions [391]. Testing these ideas could be carried out using modern optogenetics approaches (see section by Gornati and Hoebeek), but human non-invasive stimulation has provided some important clues. Long-lasting changes in motor and non-motor functions and the potential benefits in stroke, ataxia, dystonia, and tremor would suggest that cerebellar rTMS and tCDS approaches also induce changes in connected brain regions (see sections by Manto and Oulad Ben Taib, Bradnam and McCambridge, Popa and Hallett, and Timmann and Nitsche). While there is a consensus that cerebellar non-invasive stimulation is practical, relatively easy to implement in most medical institutions, and is relatively safe, there is also a consensus on several matters that must be addressed. Blind, and when possible double-blind, studies will have to be performed to solve the lack of reproducibility across some studies and consideration should be given to specificity and inter-individual differences that may also determine the effects of non-invasive stimulation. For example, one major difference between the tDCS and TMS is that at commonly used and safely applied intensities, tDCS does not trigger action potentials and therefore is less specific in its effects than magnetic stimulation. This might contribute to the lack of reproducibility between the tDCS and TMS studies. As for DBS, the mechanism of action(s) is poorly understood, and the full impact on global brain networks remain(s) unclear.

We have argued that the cerebellum should be considered as a target for neurostimulation, especially when other brain regions are, for whatever reason, not ideal. However, being that the cerebellum is in its infancy as a therapeutic neurosurgical target, perhaps to play devil’s advocate for a moment, we could ask why the cerebellum should not (yet) be considered. There is a need to understand which regions of the cerebellar cortex are best suited for different disease conditions, should stimulation paradigms take into account zones, and when should the cerebellar nuclei be considered instead? If the cerebellum is a better target in particular cases, which nucleus should be targeted? We have to consider that stimulating a given nucleus might not only result in a specific outcome because of its circuit connectivity, but also because each nucleus could have a very different composition of glutamatergic, GABAergic, and glycinergic neurons. It should also be noted that diseases such as ataxia can have developmental rearrangements in the circuitry [392], which means that stimulating a certain target might not yield the predicted outcome. Even if a desired location is theoretically ideal, what is the evidence that the electrode, in the case of DBS, will remain in place? We know from experimental studies in rodents that while tetrodes maintain stability in regions such as the hippocampus, there can be considerable drift in the cerebellum. This could be due to several factors such as curvature of the cerebellum, shape of the overlying bone and sinuses, tissue density, or its relatively weak mechanical attachment to the rest of the brain via three pairs of fiber bundles, which all could affect the proper anchoring of electrodes to the targeted region. Current ongoing studies in human stroke patients (see section by Cooperrider and colleagues) could help address many of these concerns, in addition to determining the types of electrodes that are most suitable for the cerebellum, and what the long-term impact on the tissue is. That is, what type of damage response is initiated locally within the cerebellum—or by the cerebellum and then communicated to connected regions—and are there any contraindications that arise, and how might they be dealt with by troubleshooting and modifying the approach?

Another major consideration, with its own hurdles, is if and when to use brain stimulation in pediatric patients. The sheer number of neurons and glia in the cerebellum, and its protracted developmental timetable all contribute to its high level of susceptibility to injury and disease. The diseases that affect cerebellar development are many, including well-known disorders such as ataxia (several forms), hydrocephalus, medulloblastoma, cerebral palsy, preterm birth, and ASD. Interactions and expression in gene networks are significantly altered, and as a consequence the normal dynamics of morphogenesis are abnormal. The normal dynamics of typical cerebellar development already pose challenges for predicting when it might be safe and effective to intervene with stimulation, and with the added complexity of disease-induced changes, the need for determining when and where the best stimulation targets are, becomes even greater. That is, a reasonable target at one time point during development may be inappropriate at another time point because of structural and functional changes based on neuronal migration, circuit connectivity, synaptic plasticity, and gene expression. These biological properties of the cerebellum (and for that matter, all brain regions) are core features for asking what ethical standards must be in place to consider stimulation in children, especially for invasive approaches such as DBS.

Although cerebellar stimulation is not a new idea, its focus has changed over the years. For instance, in 1809, stimulation was used to understand the role of the cerebellum in behavior, as evidenced by Rolando showing that galvanic stimulation of the cerebellum could induce movement [393]. This experimental use of cerebellar stimulation to better understand cerebellar function was carried well into the 1940s [392]. From the 1950s to the 1980s, cerebellar stimulation gained more attention (albeit controversial) for its use in the treatment of disease [156, 394–396]. There has now since been a major overhaul in the ethical standards throughout medical practice around the world, but there are still many important scientific findings and ethical lessons to draw upon from what researchers are now describing as a dark era of neurosurgery [394]. Furthermore, advances in our understanding of cerebellar development, genetics, anatomy, and electrophysiological properties provide an ever-growing number of ideas towards which one can be enthusiastic about, especially given the range of motor and non-motor functions that are now attributed to normal cerebellar function. In addition, the technical advances made in device and electrode design and neurosurgical targeting techniques should encourage a revistation to cerebellar stimulation for defined patient populations. Perhaps the firmest consensus that we have come to is that studies of cerebellar neurostimulation should proceed, but only with the sharpest critical eye for experimental and ethical standards, alternate explanations, and the mechanisms of action for how each stimulation paradigm works.

## Concluding Remarks (R.V. Sillitoe)

As we continue to unravel the many functions and anatomical connections of the cerebellum, including its unexpected roles in non-motor function and its direct links to the basal ganglia, its utility as a target for therapeutic neurostimulation will expand. As a field, we should proceed with cautious optimism that the cerebellum could be a much-needed source of corrective signals in a number of diseases. We look forward to further experimentally testing whether cerebellar cortical, cerebellar nuclear, or even cerebellar peduncular stimulation could be beneficial in ataxia, dystonia, tremor (multiple forms), epilepsy, stroke, and a growing list of disorders. Moreover, optogenetics and related approaches suggest that perhaps circuit specific stimulation could even be possible. We also look forward to further exploring the efficacy of newly developed cerebellar stimulation paradigms, such as low-intensity focused ultrasound, in disease [397]. Whatever clinical successes are achieved, we must continue to ask, how does neurostimulation work?
